# Hybrid modeling for industrial fermentation processes with an “Intra-Batch Experimental Design”

**DOI:** 10.1093/jimb/kuag014

**Published:** 2026-06-02

**Authors:** Marc Lemperle, Pedram Ramin, Julian Kager, Benny Cassells, Stuart Stocks, Krist V Gernaey

**Affiliations:** Process and Systems Engineering Center (PROSYS), Department of Chemical and Biochemical Engineering, Technical University of Denmark Søltofts Plads, DK-2800 Kgs. Lyngby, Denmark; Process and Systems Engineering Center (PROSYS), Department of Chemical and Biochemical Engineering, Technical University of Denmark Søltofts Plads, DK-2800 Kgs. Lyngby, Denmark; Process and Systems Engineering Center (PROSYS), Department of Chemical and Biochemical Engineering, Technical University of Denmark Søltofts Plads, DK-2800 Kgs. Lyngby, Denmark; Novonesis, Fermentation Pilot Plant, Krogshøjvej 36, 2880 Bagsværd, Denmark; Novonesis, Fermentation Pilot Plant, Krogshøjvej 36, 2880 Bagsværd, Denmark; Process and Systems Engineering Center (PROSYS), Department of Chemical and Biochemical Engineering, Technical University of Denmark Søltofts Plads, DK-2800 Kgs. Lyngby, Denmark

**Keywords:** hybrid modeling, industrial fermentation, oxygen transfer rate, experimental design

## Abstract

Successful development of a predictive digital twin or digital shadow enabling improved batch planning and process optimization of industrial fungal fermentation relies on the fidelity of oxygen transfer rate modeling. Such models depend, among other factors, on viscosity. Traditional process models use mechanistic approaches to describe the apparent viscosity but face challenges due to its inherent complex behavior, requiring many assumptions and often relying on tedious offline rheological measurements. This article presents a novel model development framework, which significantly reduces associated experimental costs and eliminates the need for offline rheological measurement. First, a method for developing a model for the oxygen mass transfer coefficient (*k*_L_*a*) at pilot scale is presented, reducing experimental effort from nine to two fermentations while achieving an *R*² of 0.92 with online data compared to an *R*² of 0.67 with offline data. Alongside the mechanistic model, which links biological yields to the fungal growth rate, three machine learning algorithms were evaluated as a data-driven soft sensor for predicting online viscosity across different strains and scales. The outcome is a hybrid model that requires less manual lab work for its development while predicting the dynamics of six pilot-scale fermentations under a variety of operating conditions with a modest improvement in accuracy. Results emphasize the importance of integrating online sensor technologies into mathematical model development and highlight their role in advancing data-driven methods. By lowering the costs and efforts of model development, this study contributes to the long-term vision of automated model development for industrial applications.

**One-sentence summary** This study presents a novel hybrid modeling approach that minimizes experimental efforts by replacing offline viscosity measurements with online dynamic viscosity data to predict oxygen transfer for industrial fungal fermentation processes at a 550 L pilot scale, enabling more time, and cost-efficient bioprocess model development.

## Graphical Abstract

**Graphical Abstract:**
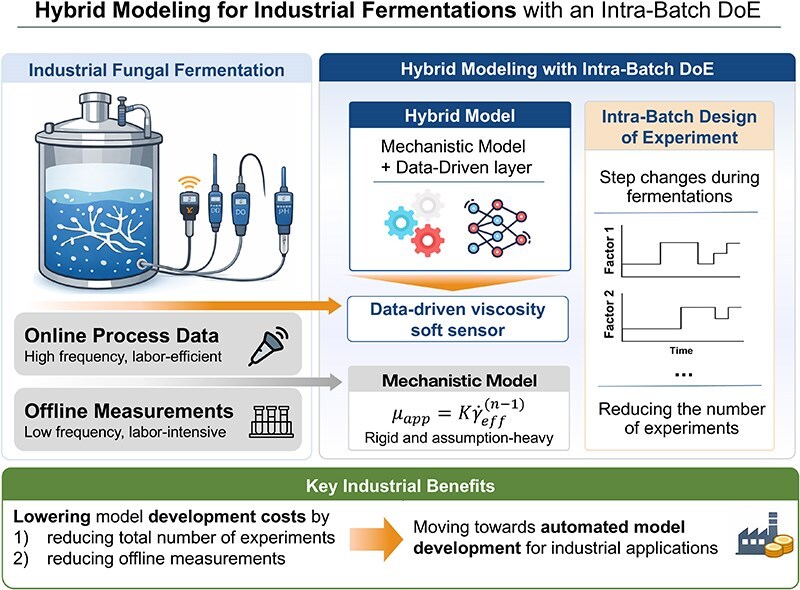

## Nomenclature

*CO_OL_*
: Oxygen concentration in the liquid phase $( {\frac{{{\rm mol}\ {{\rm O}_2}}}{{\rm kg}}} )$

$CO_{OL}^*$

: Oxygen saturation concentration $( {\frac{{{\rm mol}\ {{\rm O}_2}}}{{\rm kg}}} )$

${{F}_{i{{n}_{\textit{feed}}}}}$

: Substrate feeding rate $( {\frac{L}{h}} )$

${{F}_{ou{{t}_{\textit{evap}}}}}$

: Water evaporation rate $( {\frac{{Kg}}{h}} )$
*K*
: Consistency index (Pa*s*^n^*)
*k_L_*
: Liquid mass transfer coefficient $( {\frac{m}{s}} )$
*k_s_*
: Metzner and Otto or (shear rate) constant
*k_L_a*
: Volumetric oxygen mass transfer coefficient $( {\frac{1}{h}} )$
*OUR*
: Oxygen uptake rate $( {\frac{{{\mathrm{kg}}}}{h}} )$
*CER*
: Carbon evolution rate $( {\frac{{{\mathrm{kg}}}}{h}} )$
*AUR*
: Ammonia addition $( {\frac{{{\mathrm{kg}}}}{h}} )$
*n*
: Flow behavior index (−)
*N*
: Impeller speed (rpm)
*OTR*
: Oxygen transfer rate $( {\frac{{{\rm mol}\ {{\rm O}_2}}}{{{\rm kg}*h}}} )$
*P*_total_
: Power input from agitation and aeration (kW)
*r_p_*
: Specific production rate $( {\frac{{gP}}{{gX*h}}} )$
*S*
: Substrate concentration$\ ( {\frac{{gS\ }}{{\rm kg}}} )$
*v_g_*
: Superficial gas velocity $( {\frac{m}{s}} )$
*W*
: Liquid weight in the vessel (kg)
*Y_SX_*
: Observed yield coefficient of biomass per substrate $( {\frac{{gX\ }}{{gS}}} )$

${{Y}_{{{\rm O}_2}{{X}}}}$

: Observed yield coefficient of biomass per ${{\rm O}_2}{\mathrm{\ }}( {\frac{{gX\ }}{{{\rm mol}\ {{\rm O}_2}}}} )$

${{Y}_{{{\rm CO}_2}{X}}}$

: Observed yield coefficient of biomass per ${{\rm CO}_2}{\mathrm{\ }}( {\frac{{gX\ }}{{{\rm mol}\ {{\rm CO}_2}}}} )$

${{Y}_{{\mathrm{NH}}_4^ + {{X}}}}$

: Observed yield coefficient of biomass per ${\mathrm{NH}}_4^ + $  $( {\frac{{gX\ }}{{mol\ {\mathrm{NH}}_4^ + }}} )$
*X*
: Biomass concentration $( {\frac{{gX\ }}{{\rm kg}}} )$
*a_I_*
: Product inhibition coefficient $( {\frac{1}{h}} )$
*b_I_*
: Product inhibition coefficient $( {\frac{{gP\ }}{{kg*h}}} )$
*I*
: Product inhibition $( {\frac{{gP\ }}{{kg*h}}} )$

## Greek letters

μ
: Biomass specific growth rate $( {\frac{1}{h}} )$
*μ*_max_
: Maximum specific growth rate $( {\frac{1}{h}} )$
*μ*_app_
: Apparent viscosity (Pa*s)
*μ*_online_
: Online viscosity (mPa*s)

## Introduction

Filamentous fungi are widely used in biotechnological processes to produce commodity chemicals (e.g., citric acid), pharmaceuticals (e.g., penicillin), or biological solutions such as enzymes (e.g., amylases) (Li et al., 2000; Stocks, 2013; Vishwanatha et al., 2010). Among several advantages, fungal strains simply offer a higher production yield of those compounds compared to most other types of cells, e.g., bacteria or mammalian cells (Villadsen et al., 2011). It is furthermore well known that fungal fermentation processes are characterized by an increasing viscosity over time, which is caused either by increased cell concentration, switches in microbial morphology, or product excretion of the cells (Bodie et al., 2021; McNeil & Harvey, 1993). The increasing viscosity turns out to be a significant limiting factor of mixing and oxygen mass transfer (OTR) in the bioreactor. Given the well-documented influence of viscosity on mass transfer in fungal fermentations, this study considers it a necessity to incorporate viscosity in a *k*_L_*a* correlation (Garcia-Ochoa & Gomez, 2009; Van ’t Riet, 1983). Viscosity, which describes a fluid’s resistance to deformation or flow, can be measured using different principles, leading to challenges in comparability. Dynamic viscosity is an intrinsic, shear-rate-independent measure of internal friction, whereas apparent viscosity is shear-rate-dependent and reflects how non-Newtonian fluids respond under specific conditions (Cascaval et al., 2007; Kemblowski & Kristiansen, 1986; Kivilcimdan Moral & Sanin, 2012). In this study, dynamic viscosity is measured with an online probe and is hereafter referred to as online viscosity.

Like many other gases, oxygen has a relatively low solubility in aqueous solutions, leading to a limited maximum driving force, further constraining the achievable OTR (Villadsen et al., 2011). However, stable and sufficient OTR is crucial in aerobic fermentation processes due to its importance for cell growth and production of the targeted product. The gas-liquid mass transfer in bioprocesses is influenced mainly by the hydrodynamics in the bioreactor. The energy dissipation from the operational conditions (agitators, aeration, etc.), the geometrical parameters of the bioreactor, and the physicochemical properties of the culture broth are among the most influential factors of the OTR (Garcia-Ochoa & Gomez, 2004, 2009). A rule of thumb implies that the more viscous the fermentation broth is, the more costly it becomes to achieve sufficient mass transfer to produce a uniform, well-mixed cell suspension (Gibbs et al., 2000).

The design of experiments (DoE) principles serve, among other purposes, as an essential bridge between experimental research and mathematical modeling. With the aim of maximizing the information gained while minimizing experimental costs, DoE provides a range of methods, from simple one-factor-at-time (OFAT) approaches to more advanced methods like model-based experimental designs or intensified DoE (von Stosch & Willis, 2017). By systematically investigating process variables, DoE provides efficient solutions to optimize experimental workflows and mitigate the financial constraints associated with research and development.

For instance, the oxygen transfer rate (OTR), which is crucial for bioprocess modeling, scale-up, reactor design, and overall bioreactor performance, has been investigated in the past using DoE principles (Albaek et al., 2011). Numerous studies have been conducted to improve the understanding, optimization, and prediction of the OTR in fermentation processes, reflecting its fundamental role in ensuring efficient oxygen availability and microbial productivity (Aroniada et al., 2020; Garcia-Ochoa & Gomez, 2009; Zhu et al., 2001). However, these investigations typically depend on comprehensive datasets generated from numerous labor-intensive fermentations to thoroughly explore the experimental space. Due to the complexity of fermentation processes, creating a qualified data set, even with a suitable DoE approach, involves a considerable investment of time, resources, and costs, which often imposes a significant limitation on development of bioprocess models. Thus, the lack of rapid, cost-effective, and information-rich experiments is one of the major bottlenecks in the development of mathematical models in industrial biotechnology (Cruz Bournazou et al., 2017).

Although one can find many different empirical correlations to estimate the volumetric OTR coefficient (*k*_L_*a*) in the literature, for both Newtonian (Costa & Kooyman, 1981; Perez & Sandall, 1974; Schlüter & Deckwer, 1992) and non-Newtonian fluids (Nishikawa et al., 1974; Yagi & Yoshida, 1975), there is, however, no final agreement on which is the most suitable correlation for non-Newtonian fermentation broths. However, there seems to be a general acceptance that effects of viscosity on the OTR must be considered (Garcia-Ochoa & Gomez, 2009). Accordingly, if a bioprocess model contains a viscosity-dependent *k*_L_*a* model, it consequently requires a model to predict viscosity. Traditionally, mechanistic viscosity models have been developed based on offline measurements, such as biomass concentration and rheological analysis, both of which are labor-intensive and time-consuming. Given the non-Newtonian nature of fermentation broths, viscosity estimations have relied on correlations between power law parameters and process variables, such as the biomass concentration. These correlations are subsequently extrapolated to the average shear rate within the bioreactor. However, this approach introduces significant variability in viscosity predictions due to the dependency on measurement techniques and the shear rate ranges considered, leading to inconsistencies across different experimental setups (Kemblowski & Kristiansen, 1986; Leduy et al., 1974). Consequently, the development of mechanistic models describing the apparent viscosity is rarely and poorly tackled due to the above-mentioned difficulties (Grant Allen & Robinson, 1990; Wucherpfennig et al., 2010).

In complex scenarios where reliable predictions cannot be made using mechanistic models, new digital technologies such as artificial intelligence (AI) or machine learning (ML) are emerging as a promising method to address persistent challenges to support more efficient bioprocess modeling (Mowbray et al., 2021). The approach to overcome current challenges in mechanistic modeling is not completely new and has been applied before (Albino et al., 2024). ML models like artificial neural networks (ANNs) (Aehle et al., 2010; Gnoth et al., 2010; Preusting et al., 1996; Roubos et al., 1999) or partial least squares (PLS) (Lopez et al., 2020) have been used to estimate fermentation states such as biomass, substrate, and product concentration based on online fermentation data. Previously, work has been carried out regarding the use of decision tree-based gradient boosting algorithms (GBA), which require less computational effort and are referred to as the preferred solution for data-driven models when processing tabular data, representing a reasonable alternative to ML models such as ANNs (Garcia-Ochoa & Gomez, 2004, 2009). Rydal et al. and Tokuyama et al. both successfully employed a GBA model for the development of a hybrid soft sensor (Rydal et al., 2024; Tokuyama et al., 2021). Prior advancements in fermentation technology have enabled a more detailed characterization of process states and parameters through the integration of online data, offering significant improvements in process modeling (Kager et al., 2018).

This study tackles the challenges related to developing cost-efficient models for industrial bioprocesses involving high-density fungal fermentations by offering innovative solutions, i.e., reducing experimental effort and enhancing predictive accuracy. First, a novel method for estimating *k*_L_*a* correlations is introduced, which significantly reduces the experimental efforts. This approach allows for the construction of an empirical *k*_L_*a* correlation using data from only two fermentation processes, enabling comprehensive coverage of the design space through step changes. This methodology, referred to as “Intra-Batch Experimental Design”, offers a more cost-effective and time-efficient alternative to the conventional execution of DoE strategies, where typically one operating condition is maintained for the entire batch. Second, the study improves the *k*_L_*a* correlation by including online viscosity measurements, addressing limitations associated with offline rheology measurements. By integrating online viscosity into the *k*_L_*a* model, the study presents a superior correlation that enhances model accuracy and reliability. Third, a hybrid modeling approach is presented, in which online viscosity, which is part of the *k*_L_*a* model, is predicted by a data-driven based soft sensor. By comparing the overall online viscosity prediction performance of different ML approaches, i.e., ANN, PLS, and a light gradient boosting machine (LGBM) model, the LGBM is selected and implemented in the hybrid model framework. The ML models are trained exclusively on historical online data that can be represented from the mechanistic model, with training data from both pilot- and production-scale fermentations, as presented in detail in the Supplementary Material.

The hybrid model represents the first pivotal step toward developing an accurate and less experimentally expensive digital twin for industrial purposes, including functions such as the prediction of the necessary reactor start-fill volumes to achieve a targeted end-fill, optimization of energy costs in enzyme production, and model-based control of the system. To enable such applications, a sufficiently accurate predictive model is required. The mechanistic metabolic part of the fermentation model is intentionally designed to be as simple as possible, incorporating a few key modifications and assumptions compared to previously published models of a similar fungal process (Albaek et al., 2011; Mears et al., 2017). Given the challenges associated with modeling apparent viscosity, integrating ML offers a practical solution for improving prediction accuracy and is well aligned with the overarching goal of automating pilot-scale model development by reducing costs and labor (Rønnest et al., 2011).

## Materials and methods

### Experimental data

#### Dataset for the development of the mechanistic bioprocess model

The mechanistic model is developed using a dataset consisting of six fermentation processes, producing one industrial enzyme (referred to as Product A), as summarized in Table 1. The fermentations were operated at Novonesis, Bagsværd (Denmark). The primary goal of these fermentations was to develop and investigate (i) a shortcutting approach for estimating a *k*_L_*a* correlation using both online and offline viscosity data and (ii) to develop and calibrate a mechanistic model for the biological system. The six fermentations varied in agitation, aeration, and head pressure to fulfill those two goals. The six fermentations can be classified in two groups. Group 1: three fermentations with step changes in the before mentioned process parameters throughout the fermentation, here referred to as “step-change fermentation 1 to 3”, and group 2: three fermentations maintaining constant setpoints, referred to as “constant fermentation 1 to 3”. All fermentation processes were carried out in a dissolved oxygen (DO)-controlled fed-batch mode, as described by Albaek et al. (2012). In this setup, a defined DO concentration is maintained by controlling the substrate feed into the reactor, where the carbon source becomes the rate-limiting substrate. Standard online measurements, such as pH, temperature, pressure, agitator power, reactor mass, airflow, partial pressure of oxygen, feed rate, off-gas, and ammonia flow rate, were collected. Additionally, online viscosity data was gathered using the XL7 vibrational viscometer probe (Hydramotion Ltd., n.d.). The XL7 probe measures online viscosity by correlating the power consumption of its oscillating shaft with the dynamic viscosity of the fluid. It operates at a shear rate of 1,200 1/s, with the manufacturer claiming that operating conditions, such as stirrer speed, have no influence on the measured viscosity. The online viscosity data was only available for three of the six fermentations in the dataset, which were used for the *k*_L_*a* development and validation. Further data was obtained through offline sampling of cell dry weight (CDW) and rheological characterization, which followed the same procedures as outlined in the literature (Nadal-Rey, 2020).

**Table 1:** Overview of the fermentation batches for the mechanistic and data-driven model development and validation. The specific order of the step changes pattern, including the first phase, which denotes the outgrowth phase of the batch. The first sign in the pattern order represents the agitation, the second the pressure, and the last the aeration. The minus represents the minimum, the plus the maximum, and the 0 the center point for each of those factors. The constant fermentation processes are performed without any online measurement of viscosity due to plant capacity limitation.

Fermentation name	Main purpose	Description	Online viscosity	Offline data	Product	
Step-change fermentation 1	*k*_L_ *a* correlation estimation	Pattern order: (—) (-+-) (-++) (+-+) (++-) (000) (-++)	present	CDW, rheology, product, substrate	Product A	**Dataset for mechanistic model development**
Step-change fermentation 2	*k*_L_ *a* correlation estimation	Pattern order: (+++) (+–) (–+) (—) (000) (+–) (+-+)				
Step-change fermentation 3	Validation of *k*_L_*a*	Pattern order: (000) (–+) (+-+) (+–) (++-) (-+-) (000)				
Constant fermentation 1	Biological model development	Constant setpoints for agitation, aeration and pressure (+++)	Not present			
Constant fermentation 2	Biological model development	Constant setpoints for agitation, aeration and pressure (000)				
Constant fermentation 3	Biological model development	Constant setpoints for agitation, aeration and pressure (-—-)				
27 fermentations	Training of the data-driven online viscosity model	A more detailed description in Supplementary Table S2	present	None	See Supplementary Table S2	**Dataset for data-driven model development**
Step-change Fermentation 3	Validation of the online viscosity model	See above.			Product A	
Product A—fermentation 8	Validation of the online viscosity model	Similar to the constant fermentation 1 but outside the actual experimental work, conducted in the fermentation pilot plant				
Product B—fermentation 1	Validation of the online viscosity model	Different product than all but one other fermentation appearing in the machine learning training data set.			Product B	

#### Dataset for the development of the ML online viscosity model

Online data from 30 fermentations, conducted at both production and pilot plant scales at Novonesis, was used to train and validate the ML online viscosity models. Many of the 30 fermentations varied in key features such as the produced product and reactor volumes, resulting in 13 different products and a bioreactor volume ranging between 550 L to full production scale. Furthermore, the selected fermentation processes are constrained to the utilization of two different fungal hosts, including *Aspergillus* and *Trichoderma* strains. Approximately two-thirds of the data originates from pilot-scale batches, while the remaining one-third is derived from production batches. For model training, 27 out of the 30 fermentations were used, while the remaining three fermentations were excluded from the trainings set for further validation. Notably, two fermentation processes (step-change fermentation 1 and 2) used to fit the *k*_L_*a* correlation were also part of the training set, and the final validation fermentation for the *k*_L_*a* correlation (step-change fermentation 3) served a dual purpose as a second validation for the online viscosity prediction. Table 1 reflects the intrinsic diversity of the industrial dataset. The data-driven model was designed to predict online viscosity based on input features in a regression framework.

The online viscosity profiles from all 30 fermentations as well as a table with a more detailed batch overview, including the different products associated with the fermentations (product A, B, etc.), can be seen in the Supplementary Material (Table S2 and Figure S4). The three fermentations used for validation are summarized as follows: Two of the fermentations produced product A, which appears 9 times in the dataset, while the third fermentation produced product B, which occurs only once in the dataset and was conducted at full scale. In Table 1, these validation fermentations correspond to step-change fermentation 3 (product A), fermentation 8 (product A), and fermentation 1 (product B).

All online data was smoothed, if not stated otherwise, by applying a rolling window method with a specific window size of 5, adjusting the target value with the average of the four previous values. Outliers have been removed by applying the *z*-score method and a *z*-score threshold of 3 (Aggarwal et al., 2019). Starting from a data series of approximately 800–1,300 data points, each fermentation process dataset for the ML model was interpolated to a length of 1,500 data points by linear interpolation to facilitate more convenient data handling.

## Methods

### Development of the empirical *k*_L_*a* correlation

**Figure 1:**
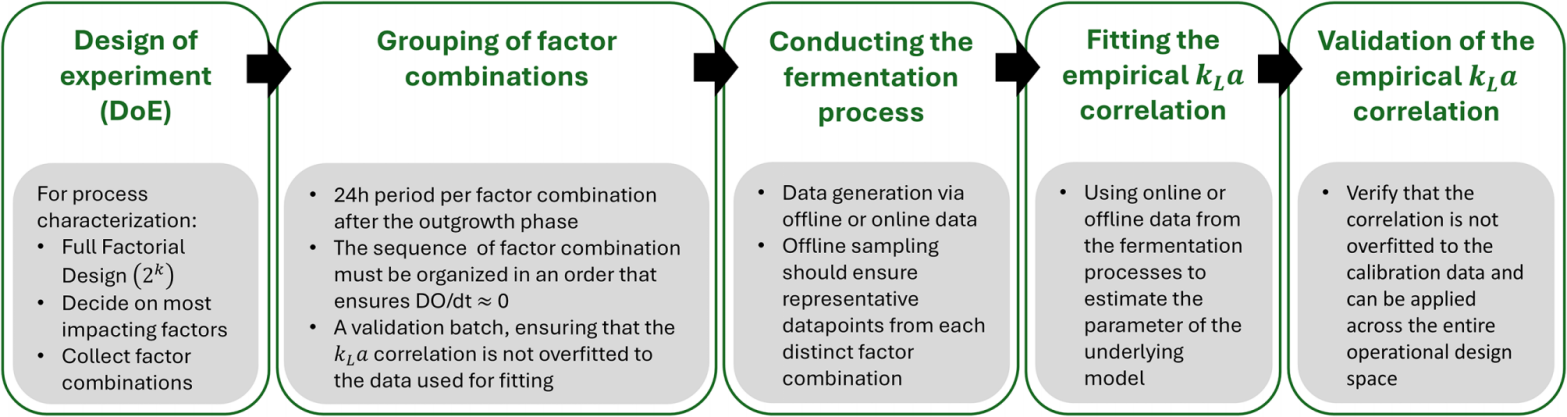
Schematic workflow of the “Intra-Batch Experimental Design” to reduce experimental work while generating a comprehensive dataset for parameter estimation of an underlying *k*_L_*a* correlation.

The “Intra-Batch Experimental Design” approach to generate a comprehensive dataset for developing an empirical *k_L_a* correlation relies on maintaining a sufficiently stable DO level during the step changes. Although in most industrial fermentations, biomass growth and viscosity increase during the process, this does not affect the validity of this approach as it is reflected as a parameter in the *k*_L_*a* correlation. It is important to emphasize that one is not fitting an entire biological model, but the physical OTR, which is kept in a steady state throughout the fermentation (Garcia-Ochoa & Gomez, 2004, 2009). Figure 1 illustrates the structured workflow, designed to reduce the number of fermentations while ensuring the generation of a comprehensive data set for parameter estimation of the underlying *k*_L_*a* correlation. The process begins by selecting an appropriate DoE, which is guided by the objective of the study. For process characterization, this leads to the use of a full factorial experimental design. This experimental design systematically investigates the operating space by focusing on the corner points, at which the minimum and maximum values of the factors are located, thus ensuring that the broadest range of process conditions is considered. In addition, the inclusion of a center point, where all factors are set to their median value, increases the statistical robustness of the data set. The design consists of 2^*k*^ + 1 experimental conditions, with *k* denoting the number of factors, providing a reliable basis for parameter estimation (Antoy, 2014).

The next critical step is to group factor combinations to minimize the experimental workload by combining multiple factor combinations into a single fermentation process. A 24-hr period for each factor combination to allow for sufficient system response time and comprehensive data collection is recommended but depends on the reactor and biological systems’ ability to respond to the changes. The transition between each combination of factors should be considered in respect to expert knowledge of the system and the specific fermentation. A ramp period of 10–12 hr proved effective for the use case presented in this work. To maintain experimental robustness, the sequence of factor combinations must be carefully selected to ensure that the bioreactor system is able to adjust the feed rate accordingly to the changes in OTR, ensuring a sufficiently stable DO level. In this phase, a fermentation for validation purposes is recommended to verify the *k*_L_*a* correlation. The next three phases involve performing the planned fermentations with an organized data collection for each factor combination, fitting the empirical *k*_L_*a* correlation to the collected data and finally validating the model against the experimental results, as illustrated in Figure 1. The *k*_L_*a* value used for fitting and validating the correlation can be determined experimentally using the direct method (Villadsen et al., 2011). The low magnitude of the rate of change in DO concentration $( {\frac{{dDO}}{{dt}}} )$, a direct consequence of the inherently low solubility of oxygen in water, supports the assumption that OTR ≈ OUR. This assumption is most accurate when DO is held constant, but even during the outgrowth phase, where DO decreases, the resulting $\frac{{dDO}}{{dt}}$ remains very small relative to the oxygen uptake rate. When expressed in the same units, e.g., $\frac{{\textit{mmol}}}{{kg*h}}$, one can see that the errors introduced into the *k*_L_*a* correlation by including these early data points are below 1% (Villadsen et al., 2011). For this reason, the *k*_L_*a* values obtained during the outgrowth phase are still considered reliable and are included in the analysis. Henry’s law is used to calculate the saturated DO concentration $( {CO_{OL}^*} )$ based on the composition of the inlet gas (20.94% O₂) and the actual pressure conditions in the reactor, considering head pressure, atmospheric pressure and hydrostatic pressure. The actual DO (*CO_OL_*) is measured by a DO probe, which enables one to determine *k*_L_*a*, as presented in equation 1 (Van ’t Riet, 1983; Villadsen et al., 2011).


(1) \begin{eqnarray*}
{{k}_{\mathrm{L}}}{{a}} = \frac{{{\mathrm{OUR}}}}{{CO_{OL}^* - C{{O}_{OL}}}}.
\end{eqnarray*}

The presented structured approach in Figure1 ensures robust data generation of the entire DoE and minimizes experimental workload for the estimation of the *k*_L_*a* correlation, referred to as “Intra-Batch Experimental Design”.

### Mathematical description of the mechanistic model

As presented in Albaek et al. (2011), the fed-batch fermentation process reaction can be simplified to the following reaction in equation 2.


(2) \begin{eqnarray*}
\textit{Substrate} + {{Y}_{S{{O}_2}}}*{{O}_2}\to {{Y}_{SX}}*\textit{biomass} + {{Y}_{SP}}* \textit{product} + {{Y}_{SC{{O}_2}}}*C{{O}_2}.
\end{eqnarray*}

### Mass transfer prediction

The OTR can be described by equation 3:


(3) \begin{eqnarray*}
OTR = \ {{k}_{\mathrm{L}}}a*\left( {CO_{OL}^* - C{{O}_{OL}}} \right).
\end{eqnarray*}

Equation 3 consists of *k*_L_, the liquid side mass transfer coefficient $( {\frac{m}{s}} )$, the surface area of the gas bubbles contained in a unit volume of liquid, the liquid interface $a( {\ \frac{{{{m}^2}}}{{{{m}^3}\ }}} ),$ and the driving force between the maximum saturated DO concentration ($CO_{OL}^*$) at the interface between the gas bubbles and the liquid and the actual DO concentration (*CO_OL_*) inside the liquid. Therefore, as is generally understood, the transport limitation occurs between the gas bubbles and the liquid layer around the bubbles (Van ’t Riet, 1983; Villadsen et al., 2011).

In literature, many different empirical correlations for the volumetric oxygen transfer coefficient *k*_L_*a* are proposed (Garcia-Ochoa & Gomez, 2004). Most correlations for non-viscous fluids are dependent on the specific power input $(\frac{{P}_{{\rm total}}}{V} ),$ which includes both the shaft power, the power produced by the isothermal expansion of the gas as well as the superficial gas velocity (*v_s_*). Additionally, the apparent viscosity (*μ*_app_) is considered for viscous fluids as presented in equation 4 (Albaek et al., 2011; Cooke et al., 1988; Garcia-Ochoa & Gomez, 2009; Ke et al., 2017).


(4) \begin{eqnarray*}
{{k}_{\mathrm{L}}}{{a}} = {\mathrm{C\ *}}{{\left(\frac{{P}_{\mathrm{total}}}{V} \right)}^a}*v_s^b*\mu _{{\rm app}}^c.
\end{eqnarray*}

The apparent viscosity in equation 5 can be determined by offline measurements and data fitting to the power law model, as most fermentation broths are to be considered shear-thinning. *K* represents the consistency index (Pa*s*^n^*), and *n* the flow behavior index ( − ), which are modeled as described in Albaek et al. (2011).


(5) \begin{eqnarray*}
{{\mu }_{\rm app}} = K*\mathop {\dot\gamma} \nolimits _{\rm eff}^{\left( {n - 1} \right)}.
\end{eqnarray*}

In equation 5, the effective shear rate of the fermentation vessel (${\dot \gamma}_{\rm eff}$) is empirically correlated, e.g., by the Metzner and Otto approach. *k_s_* represents the Metzer and Otto constant, specific for the impeller type and vessel geometry used for all fermentations as in Albæk (2012) and *N* represents the agitation speed (rpm), as defined in equation 6 (Pedersen et al., 1993; Petersen et al., 2008).


(6) \begin{eqnarray*}
{\dot \gamma }_{\rm eff} = {k}_{s}*N.
\end{eqnarray*}

The apparent viscosity can be calculated based on the ratio between the shear stress and the effective shear rate.

Due to challenges in estimating the apparent viscosity in a bioreactor as described before and seen in several studies (Albaek et al., 2011; Mears et al., 2017), a novel approach is proposed by replacing the apparent viscosity of equation 4 with the data generated by an online viscosity probe, giving equation 7. The fit of the new proposed model is compared against the conventional model, followed by elaborating on the advantages and disadvantages.


(7) \begin{eqnarray*}
{{k}_{\mathrm{L}}}{{a}} = {\mathrm{C\ *}}{{\left(\frac{{P}_{\mathrm{total}}}{V} \right)}^a}*v_s^b*\mu _{\mathrm{online}}^c.
\end{eqnarray*}

The fed-batch fermentation is modeled based on mass balances and consists of a set of ordinary differential equations for the mass of the fermentation broth, the concentrations of CDW (X), substrate (S), dissolved oxygen (DO), and product (P) inside the fermentation broth.

The total mass of the culture broth (W) (kg) is modeled by considering the evaporation rate of water ${{F}_{ou{{t}_{\textit{evap}}}\ }}$as well as the oxygen uptake rate *OUR* (equation 13), carbon evolution rate *CER* (equation 14), and ammonia uptake rate *AUR* *CER* (equation 15), in $\frac{{kg}}{h}$ units, as can be seen in equation 8. ${{F}_{ou{{t}_{\textit{evap}}}\ }}$is calculated as in Mears et al. (2017), assuming that the exhaust gas is saturated with water (Mears et al., 2017). Equations 9, 10, and 11 represent the rate of change of CDW, substrate, and DO, respectively, which is modeled as in Albaek et al. (2011). The product formation model includes an inhibiting term I that depends linearly on the product itself and its parameter *a_I_* and *b_I_*, as can be seen in equation 12.


(8) \begin{eqnarray*}
\frac{{dW}}{{dt}} = {F}_{{in}_{\textit{feed}}} + OUR + AUR -{F}_{{out}_{\textit{evap}}} - CER,
\end{eqnarray*}

(9) \begin{eqnarray*}
\frac{{dX}}{{dt}} = \mu *X - \frac{{\frac{{d{\mathrm{W}}}}{{{\mathrm{dt}}}}}}{W}*X,
\end{eqnarray*}

(10) \begin{eqnarray*}
\frac{{dS}}{{dt}} = {{F}_{in}} - \mu *\frac{X}{{{{Y}_{SX}}}} - \frac{{\frac{{d{\mathrm{W}}}}{{{\mathrm{dt}}}}}}{W}{\mathrm{\ *}}S,
\end{eqnarray*}

(11) \begin{eqnarray*}
\frac{{dDO}}{{dt}} = {{k}_{\mathrm{L}}}a*\left( {D{{O}^*} - DO} \right) - \mu *\frac{X}{{{{Y}_{O2X}}}} - \frac{{\frac{{d{\mathrm{W}}}}{{{\mathrm{dt}}}}}}{W}*DO,
\end{eqnarray*}

(12) \begin{eqnarray*}
\frac{{dP}}{{dt}} = {{r}_p}*X - I\ - \frac{{\frac{{d{\mathrm{W}}}}{{{\mathrm{dt}}}}}}{W}{\mathrm{*P}} | I = {{a}_I}*P + {{b}_I},
\end{eqnarray*}

(13) \begin{eqnarray*}
OUR = \frac{{\mu {\mathrm{\ *}}X}}{{{{Y}_{{{O}_2}{\mathrm{X}}}}}},
\end{eqnarray*}

(14) \begin{eqnarray*}
CER = \frac{{\mu {\mathrm{\ *}}X}}{{{{Y}_{C{{O}_2}{\mathrm{X}}}}}},
\end{eqnarray*}

(15) \begin{eqnarray*}
AUR = \frac{{\mu {\mathrm{\ *}}X}}{{{{Y}_{{\mathrm{NH}}_4^ + {\mathrm{X}}}}}},
\end{eqnarray*}

(16) \begin{eqnarray*}
{{Y}_{SX}},{{Y}_{{{O}_2}{\mathrm{X}}}},{{Y}_{C{{O}_2}X}},{{Y}_{{\mathrm{NH}}_4^ + {\mathrm{X}}}} = f(\mu).
\end{eqnarray*}

#### Dynamic yields

In many fermentation studies, yields are often considered constant over the entire process time. In contrast, our approach calculates the yields dynamically by defining the “transient yield” as the yield between two consecutive data points. In this case, the two consecutive data points are taken within 24-hr intervals. This method enables a more detailed, time-resolved understanding of the fermentation yields in the process. The reason for using a 24-hr period is to mitigate the effects of measurement errors. When the yield is calculated over shorter periods of time, the differences in the state variables, such as the concentrations of oxygen, substrate, or CO₂, are relatively small. As the absolute error in the measurements (e.g., error related to CDW value) remains constant, using shorter intervals would increase the error in relation to the measurement, which would otherwise lead to unrealistic or erratic yield values. As the dynamic pattern observed in the transient yields correlates with the biomass growth rate, a linear correlation between the growth rate and each of the following yields ${{Y}_{SX}},{{Y}_{{{\rm O}_2}{{X}}}},{{Y}_{{\rm C}{{\rm O}_2}{X}}},{{Y}_{{\mathrm{NH}}_4^ + {{X}}}}$ (equation 16) was found, which is presented in the Supplementary Material (Figure S1). The final regression expressions cannot be shared for confidentiality reasons. However, it must be highlighted that the offset of those linear regressions fulfils the purpose of a constant maintenance term. Therefore, cell maintenance of substrate, O_2_ and ammonia are implemented in the biological bioprocess model.

#### Specific growth rate

It is assumed that cell growth is only dependent on the available substrate concentrations inside the fermentation broth, as all six fermentation batches are run at the same temperature and pH value. Consequently, no DO dependency is added to the growth model, which is justified by the constant DO setpoint during most of the fermentation period. Furthermore, we anticipate that drastic changes in the feed flow rate will lead to substrate accumulation in the broth, despite efforts to prevent this through the DO-control strategy. Therefore, the Monod model in equation 17 is considered a valid model for the specific growth rate. μ_*max*_ denotes the maximum specific cell growth rate, S the substrate concentration and *K_s_* the growth saturation constant for the substrate (Villadsen et al., 2011).


(17) \begin{eqnarray*}
\mu = {{\mu }_{\rm max}}{\mathrm{\ *\ }}\frac{S}{{\left( {{{K}_s} + S} \right)}}.
\end{eqnarray*}

The parameter *μ*_max_ and *r_p_* were directly determined experimentally with the measured data.

In contrast, *K*_s_, *a*_I_ and *b*_I_ were optimized toward the experimental data. Bayesian optimization is performed to minimize the normalized root mean square error (NRMSE) between model predictions and experimental data for biomass and product concentrations outside the hybrid model in an open-loop control. The optimization results in 0.0085 for *a_I_* and 0.099 for *b_I_*. The values for *μ*_max_, *r_p_*, and *K_s_* cannot be disclosed for confidentiality reasons.

### Data-driven online viscosity prediction model

The data-driven online viscosity models were trained using historical pilot plant and production data. To identify an accurate model applicable across different organisms, the performance of three ML models (LGBM, feedforward ANN, and PLS), all trained on the same dataset with identical data points and input features, was compared by validating each model on three fermentation processes that were excluded from the model training (Chen & Guestrin, 2016; Shwartz-Ziv & Armon, 2022).

The input features were selected based on the current mechanistic understanding of the most influential features toward viscosity dynamics inside the tank. Furthermore, the input features were chosen with consideration of the accuracy of the mechanistic model outputs. Scale-dependent input features, e.g., the accumulated value of ammonia, have been scaled with the start weight mass of the fermentation to receive a scale-independent correlation from the inputs to the outputs. Label encoding was used to let the models distinguish between the different organisms and product types, denoting each product or organism with a specific real value (e.g., 0 for product A, etc.) (Shah et al., 2022). Due to the high number of data points for training, a leave-one-batch-out cross-validation for validation purposes as well as a 70/20/10 approach for the model training procedure was performed (Raschka, 2018). The hyperparameters of the LGBM, such as the type of boosting, were calibrated using the scikit-optimize package forest_minimize to achieve an optimum performance of the LGBM. The feedforward ANN was trained by implementing a multilayer perceptron (MLP) regressor using scikit-learn. Hyperparameter optimization was performed using a grid search (GridSearchCV) with fivefold cross-validation on the training set, optimizing for the *R*-squared (*R*²) score. All hyperparameter and additional information about the grid search and optimization can be found in the Supplementary Material (Table S1). Prior to training, the features were standardized using StandardScaler. The PLS regression model was trained using scikit-learn. The optimal number of latent features (components) was determined using GridSearchCV with fivefold cross-validation on the training data, also optimizing for the *R*² score (Supplementary Tables S3 and S4) (Alibrahim & Ludwig, 2021; Hossain & Timmer, 2021).

In addition, the LGBM model was interpreted and optimized with the help of SHAP (SHapley Additive exPlanations) values and residual analysis. SHAP values quantify the contribution of each input feature to the model output by computing its average marginal effect across all possible feature combinations. This approach inherently accounts for interactions between multiple features, making it more informative than traditional feature importance metrics (Hammer & Holzman, 1992) (Supplementary Figure S2). Residual analysis indicates the error between the real and predicted outcome (Supplementary Figure S3). Validation of the data-driven online viscosity models after the training procedure is performed by comparing experimental online viscosity measurements with the model predictions using the experimental ML input data from the corresponding fermentation. Therefore, three fermentation processes were taken out of the training data set. The ML model with the overall lowest error is selected to predict the online viscosity of the proposed hybrid model.

### Hybrid model

In modern bioprocess modeling, hybrid approaches combining mechanistic models with data-driven ML models are increasingly recognized for their ability to improve predictive capabilities while ensuring process understanding. These models leverage fundamental mechanistic models while incorporating ML to capture complex, nonlinear behaviors that may not be fully described by first-principles equations alone.

The hybrid model presented in this study is designed to simulate fungal fermentation processes operated at Novonesis. It is intended to serve as a key component in a digital twin framework, as described in the introduction. An attempt is made to predict the outcome of fermentation processes by considering the following: (1) a representation of the main reaction equation, (2) prediction of the mass transfer, including online viscosity, and an ML approach to predict online viscosity.

Equation 18 defines the hybrid *k*_L_*a* model, which combines mechanistic principles with a data-driven component. The mechanistic part accounts for power input and superficial gas velocity, while the data-driven part incorporates an ML online viscosity prediction. These online viscosity predictions are generated from a set of ML input features represented as ${{\bf Y}} = ( {{{Y}_1},{{Y}_2},\ldots ,{{Y}_n}} )$. Since the ML model estimates the online viscosity μ_online_ originally described in equation 6, that equation can be reformulated into the hybrid representation shown in equation 18.


(18) \begin{eqnarray*}
{{k}_{\mathrm{L}}}{{a}} = {\mathrm{C\ *}}{{\left(\frac{{P}_{\mathrm{total}}}{V} \right)}^a}*v_s^b*\left( {{{f}_{\rm ML}}({{Y}_i}\mid i = 1,\ldots n} \right){{)}^c}.
\end{eqnarray*}

### Model evaluation

For the assessment of the modeled and experimental data, the Min-Max NRMSE was employed as a benchmark for comparison and evaluation. This metric scales the RMSE by the range of observed values, enabling fair comparison across different datasets. The NRMSE is calculated as defined in equation 19, where *x_i_* represents the observed value, ${{\hat{x}}_i}$ the predicted value, *N* the number of data points, and *x*_max_ and *x*_min_ the maximum and minimum observed values, respectively. Furthermore, the *R*^2^ coefficient was used to assess the degree of correspondence between the predicted and observed *k*_L_*a* values (Botchkarev, 2018).


(19) \begin{eqnarray*}
\textit{NRMSE} = \frac{{\sqrt {\frac{1}{N}\mathop \sum \nolimits_{i = 1}^N {{{({{x}_i} -\widehat {{{x}_i}})}}^2}} }}{{{{x}_{\rm max}} - {{x}_{\rm min}}}},
\end{eqnarray*}

(20) \begin{eqnarray*}
{{R}^2} = \frac{{\mathop \sum \nolimits_i {{{\left( {{{y}_i} - \widehat {{{y}_i}}} \right)}}^2}}}{{\mathop \sum \nolimits_i {{{\left( {{{y}_i} - \overline {{{y}_i}} } \right)}}^2}}}.
\end{eqnarray*}

### Computational methods

Python 3.11.9 was used for all calculations, model simulations and visualizations. The differential equations were solved with scipy.stats.solve_ivp (version 1.14.0) using the BDF method. Parameter estimation of the kinetic model parameters is performed using Bayesian optimization via the gp_minimize function from the skopt package. *k*_L_*a* correlation parameters were estimated via the Levenberg–Marquardt-based nonlinear regression implemented in scipy.optimize.curve_fit. The LGBM model was developed using the Microsoft package lightgbm (version 4.5.0) and the sciki-optimize package forest_minimize for tuning the hyperparameters.

## Results

### Fermentation data

**Figure 2:**
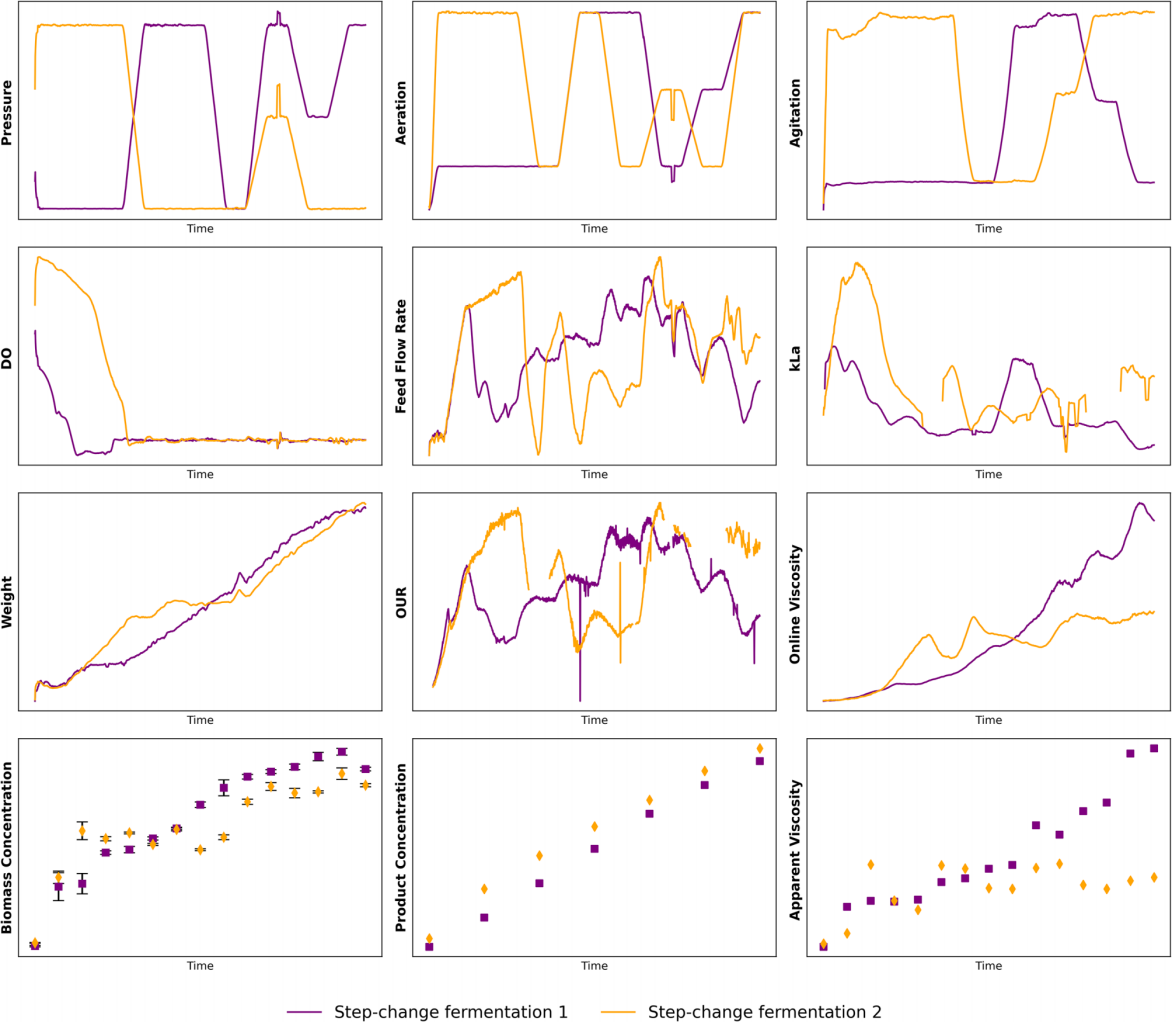
Different operational and biological parameters of the step-change fermentation 1 and 2. The process is operated with a controlled DO setpoint and substrate feeding ramps for the manipulated variable. Due to issues in the offline analysis, no measurements are available for the OUR and therefore *k*_L_*a* for two short periods in the step-change fermentation 2.

The experimental fermentation dynamics of step-change fermentation 1 and 2 are presented in Figure 2, as those two fermentations are used to fit the *k*_L_*a* correlation. As designed in the experimental plan, pressure, aeration, and agitators follow the predefined step changes (Table 1), ensuring that all corner points and the center point are covered at least once. A relatively typical DO profile demonstrates that the system can adapt to the quite radical changes in pressure, aeration, and agitation and compensate for them by adjusting the feed flow rate, which is needed for the fermentation to be used in the proposed *k*_L_*a* correlation framework. The stepwise changes lead to a dynamic *k*_L_*a* profile for the two fermentations, with sep-change fermentation 2 delivering higher *k*_L_*a* values due to the high set points of pressure, aeration, and agitation at the beginning of the fermentation. The reactor mass profile is reasonably consistent with substrate feed, ammonia, and oxygen addition.

The online viscosity probes record different profiles throughout the two fermentation processes. The online viscosity profile of step-change fermentation 1 appears more exponential, whereas step-change fermentation 2 exhibits a rapid initial increase up to the point where biological responses to the step changes occur, followed by an undulating, slightly increasing trend. The CDW measurements indicate steadily increasing values for step-change fermentation 1 with decreasing growth toward the end, as is also observed in a typical fermentation process (Albaek et al., 2011). Comparing the first three CDW data points of both fermentations, one can see the expected higher values for the fermentation with the high OTR outgrowth. Overall, CDW values of the step-change fermentation 2 suggest a decrease around the time when the fermentation is running without feed addition, signaling cell culture responses to the step changes. The product concentration indicates a more linear increase with an expected plateau toward the end for both fermentations compared to the CDW measurements. The apparent viscosity data seems to correlate briefly with the online viscosity profile. However, one should be aware that direct comparison is not possible due to the differences in measurement principles between the offline and online viscosity.

### Development of the empirical *k*_L_*a* correlation

**Figure 3:**
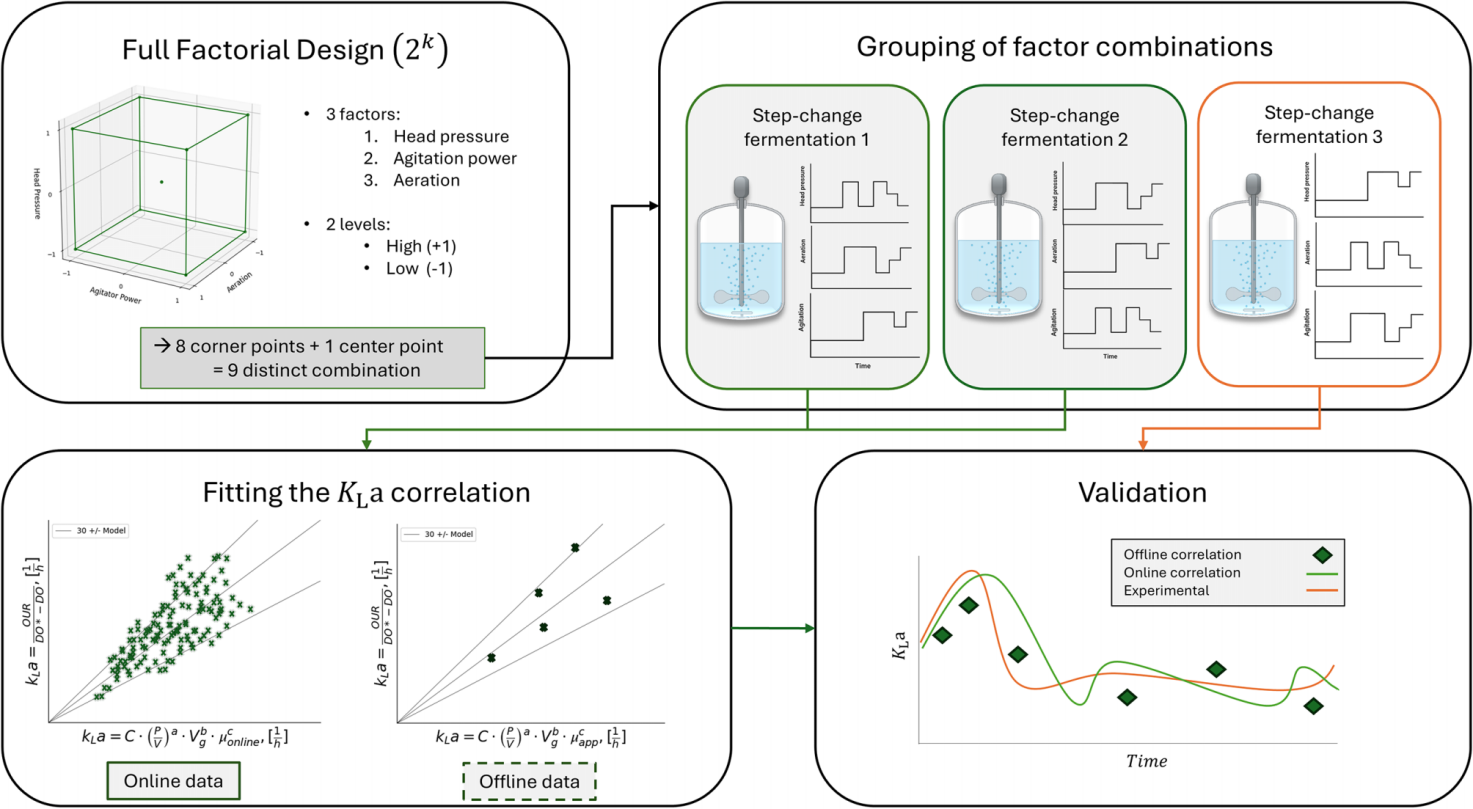
Schematic visualization of the “Intra-Batch Experimental Design”, including the full factorial design, the step-change fermentation 1, 2, and 3 as well as fitting the *k*_L_*a* correlation and validation.

Figure 3 provides a schematic overview of the “Intra-Batch Experimental Design” for estimating *k*_L_*a*, which aims to accurately characterize the oxygen transfer coefficient according to the workflow defined in Figure 1. The methodology involved a fully factorial design in which the effects of total power per volume, gas flow rate, and head pressure on *k*_L_*a* are investigated. This resulted in a 2³ factorial design that yields nine different factor combinations that were systematically distributed across two fermentation processes. To validate the results, a further fermentation process with variations in pressure, agitation, and aeration was used. Two *k*_L_*a* correlations were established: one using a data set including online viscosity measurements and the other based on offline viscosity assessment. The results of the empirical *k*_L_*a* correlations are presented in the following subsections, following the workflow suggested in Figure 3.

**Figure 4:**
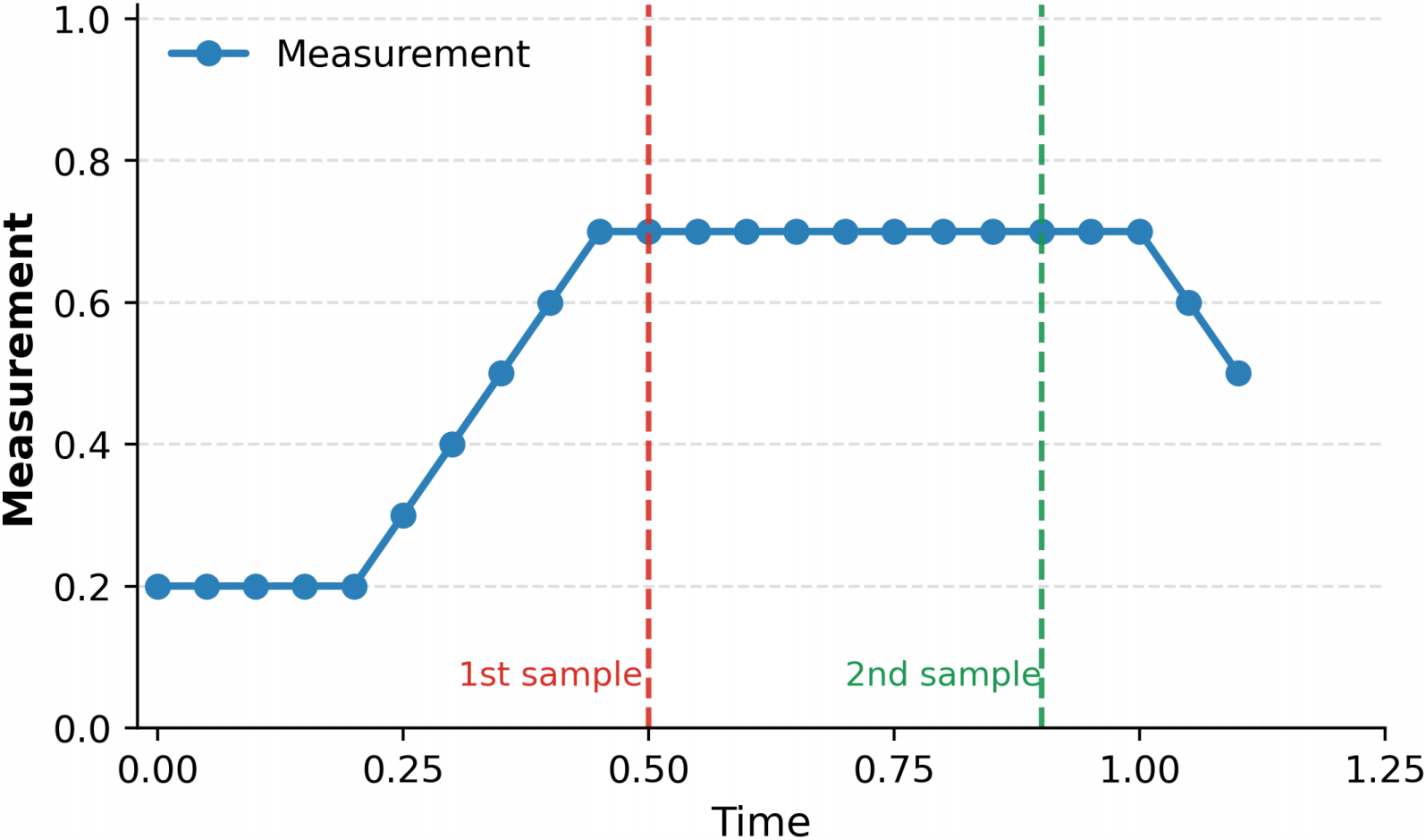
Visualization of the sampling points during each pattern.

**Grouping of factor combination:** During two fermentation processes (step-change fermentation 1 and 2), nine different distinct combinations from the full factorial design space were covered at least once over an extended period of 24 hr, including the ramps to switch between the combinations. A planned sequence of the different factor combinations was associated with an educated guess of the expected OTR, following a stepwise increase and a decrease in OTR for the fermentations 1 and 2, respectively, which can be seen in Table 1. This was chosen so that the bioreactor system can react to the changes in OTR by adjusting the feed rate accordingly, thereby ensuring a constant DO concentration. The *k*_L_*a* values are determined experimentally using the direct method, as described in the “Materials and Methods” section. A Henry’s constant value for water at the temperature of the fermentation broth was used. Offline samples for CDW and rheological measurements were taken shortly after the completion of each ramp and toward the end of those settings, as visualized in Figure 4. These points were chosen to be representative of each pattern of the operating space. Three additional offline samples were taken during the outgrowth phase. The outgrowth phase for step-change fermentation 1, 2, and 3 was operated in the settings of the first pattern, so (—), (+++), and (000), respectively.

### Fitting and validation of *k*_L_*a* correlation

**Figure 5:**
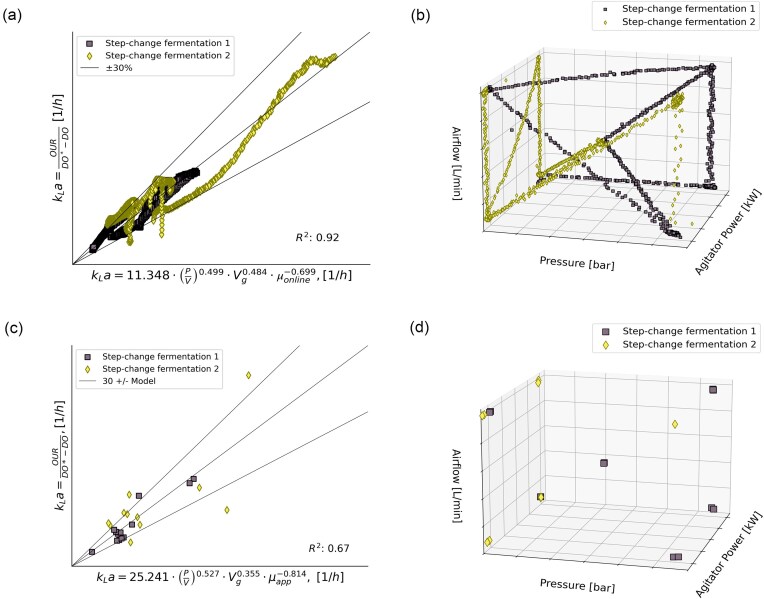
A and C: Parity plot of the measured *k*_L_*a* values and the predicted *k*_L_*a* values using online data for subplot (A) and offline data for subplot (C) of all variables, shown with the estimated parameter. B and D: Three-dimensional visualization of the experimental data in the design space, which is considered for the *k*_L_*a* correlation in subplot (A) and (C), respectively.

The equations 4 and 7 are fitted to the experimental online and offline data of the two fermentations (step-change fermentation 1 and 2) by least squares regression. The parity plot in Figure 5A between the empirical correlation and the measured *k*_L_*a* values includes a total of 1,909 data points of power per volume $( {\frac{P}{V}} )$, superficial gas velocity *v_S_* and online viscosity measurement *μ*_online_, which covers the operational space as seen in Figure 5B. The parity plot in Figure 5C presents the empirical *k*_L_*a* correlation found, including the apparent viscosity *μ*_app_ and not as the empirical online *k*_L_*a* correlation with the direct online viscosity measurement. The empirical offline correlation is estimated from the offline samples, ending up with 20 data points, which cover the operational space as seen in Figure 5D. *k*_L_*a* values below a threshold of ${{k}_{\mathrm{L}}}{{a}} \,\lt\, 10\frac{1}{h}$ were excluded from both empirical offline and online correlations, as such low values are not representative of typical operating conditions and may introduce disproportionate errors in the *k*_L_*a* calibration.

Most of the data points for the empirical online *k*_L_*a* correlation, Figure 5A are observed to be in a ± 30% range, indicating the measurement error range. Figure 5C reveals that relatively more data points lie outside the measurement error range of the offline *k*_L_*a* correlation. Furthermore, the empirical online *k*_L_*a* correlation finds a higher agreement with the experimental values while including input data for a significant higher range of *k*_L_*a* values.

The data for the empirical online *k*_L_*a* correlation indicates a better alignment with the experimental values, indicated by the higher *R*^2^ of 0.92 compared to the *R*^2^ of 0.67 for the offline data. The final parameters for the online correlation are *C* = 11.348 ± 0.732, *a* = 0.499 ± 0.007, *b* = 0.484 ± 0.013, and *c* = –0.699 ± 0.007, while the parameters for the offline correlation are *C* = 25.241 ± 23.532, *a* = 0.527 ± 0.131, *b* = 0.355 ± 0.172, and *c* = –0.814 ± 0.200.

Furthermore, the empirical online correlation benefits from lower uncertainties compared to the empirical offline *k*_L_*a* correlation, which can be seen from the error of each parameter..

**Figure 6:**
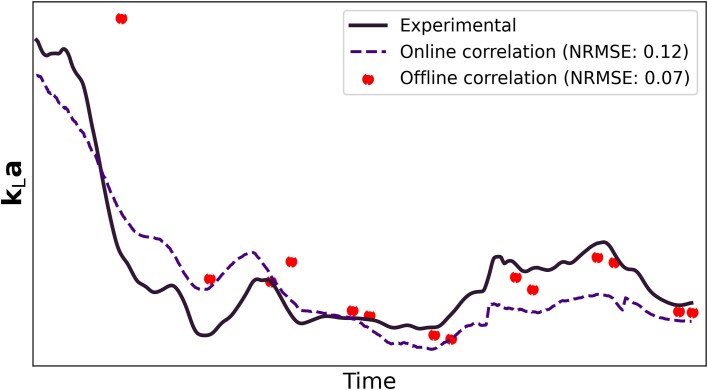
Validation of the *k*_L_*a* correlation found with OTR fermentation 1 and 2 on the Product A—fermentation 3.

The empirical *k*_L_*a* correlations found with step-change fermentation 1 and 2 are validated with the step-change fermentation 3 as visualized in Figure 6. This fermentation batch meets the validation criteria by exhibiting entirely different *k*_L_*a* dynamics, resulting from distinct step changes in the three influencing factors compared to the fermentation batches that were used for generating the data and fitting the empirical *k*_L_*a* correlations. The sequence of step changes is listed in Table 1. An NRMSE of 0.12 provides confidence in the validity of the empirical online *k*_L_*a* a correlation, which can be validated over the entire fermentation range due to the online measurements. The few predicted *k*_L_*a* values of the offline correlation find a very good agreement with the *k*_L_*a* values at those specific points with an NRMSE of 0.07.

### Data-driven online viscosity model

**Figure 7:**
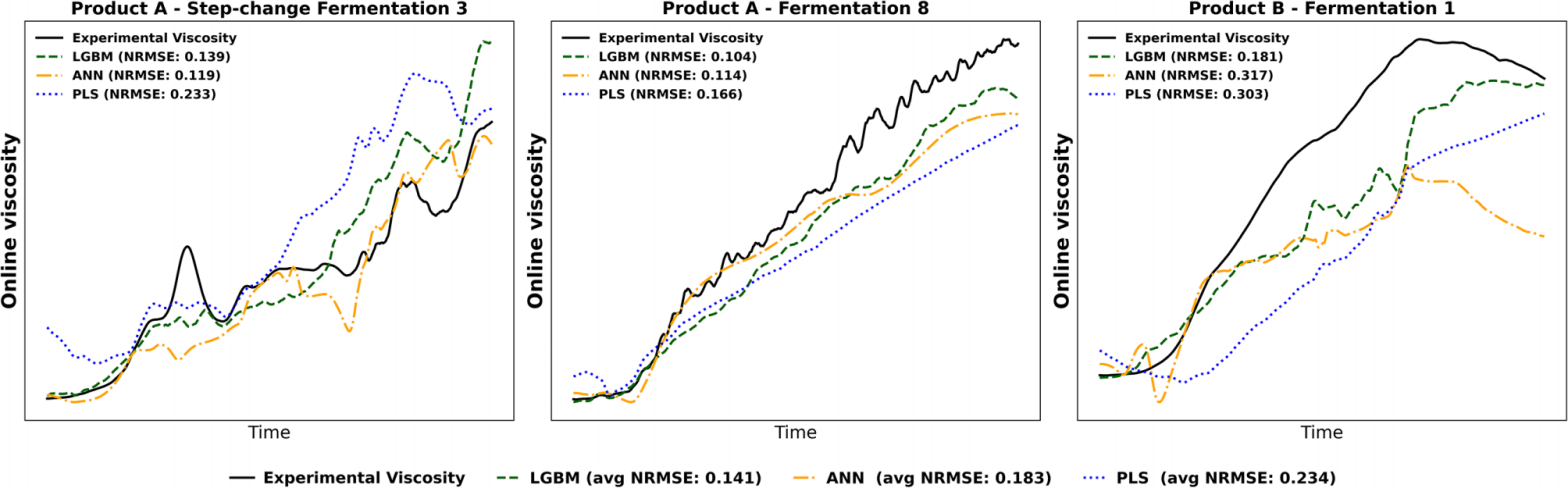
Comparison of three online viscosity profiles (experimental vs. LGBM, ANN, PLS) of step-change fermentation 3 (product A), Product A—Fermentation 1 and Product B—Fermentation 1.

**Figure 8:**
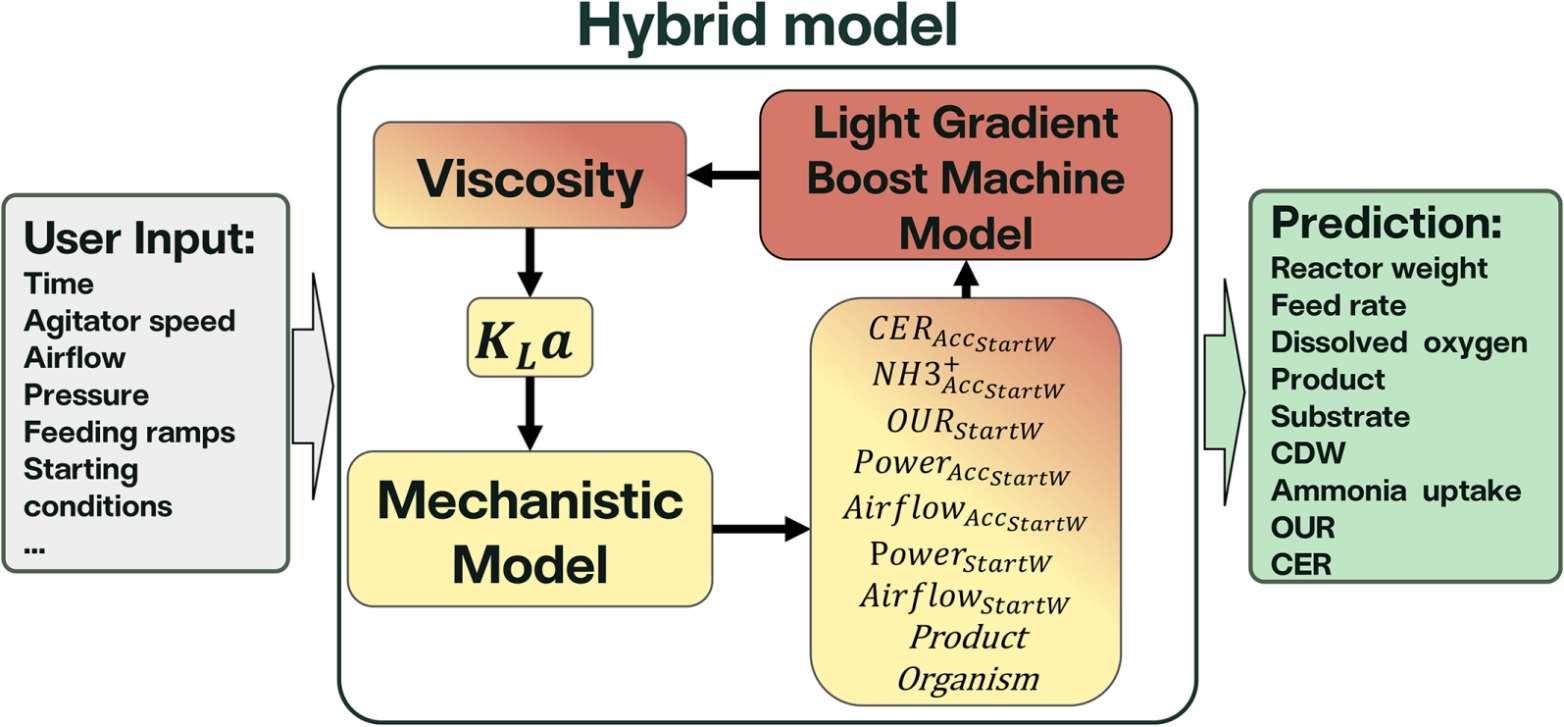
Sequential hybrid model structure, predicting the viscosity mechanistic data input variables. The interplay between the mechanistic (yellow) and machine learning model (red) as well as the user input (grey) and model output (green) is visualized by appropriate color codes as well as black arrows.

The LGBM, ANN, and PLS online viscosity model were developed and trained as described in the “Materials and Methods” section. Three, from the training data, excluded fermentation processes were used to validate and test the performance of the models, as outlined in Figure 7. The data-driven model inputs included accumulated values of CER, OUR, ammonia, power, and airflow, as well as non-accumulated values of power and airflow, along with product and organism information. These inputs are denoted as *Y*_1_ to *Y*_9_ and visualized in Figure 8. We evaluate the model’s generalizability performance on fermentations that were producing two distinct products, product A and product B. Product A is well-represented in the data set with nine fermentations, while product B is only included in the data set with one fermentation. This setup allows us to assess the model’s predictive capability for both familiar and unfamiliar fermentation processes of the trainings data. The LGBM model has an overall prediction error of 14.1%, while the ANN and PLS model predict on average with an error of 18.3% and 23.4%, respectively. A closer look reveals that the ANN does perform slightly better on the fermentation processes linked to product A but performs remarkably worse on the fermentation producing product B. These results highlight that the LGBM online viscosity model is the most robust throughout the different products.

### Process simulation

The Hybrid model (Figure 8) is developed by utilizing the online *k*_L_*a* correlation, despite the fact that the offline correlation yields a lower relative error on the validation batch (Figure 6). This choice is justified by the following: the online correlation’s higher accuracy across a broader range of *k*_L_*a* values on the calibration data (Figure 5), its lower uncertainty, and most importantly, the overarching objective to reduce the need for offline sampling and increase reliance on online data for model development for industrial applications. Consequently, the need for a mechanistic apparent viscosity model is thus eliminated.

**Figure 9:**
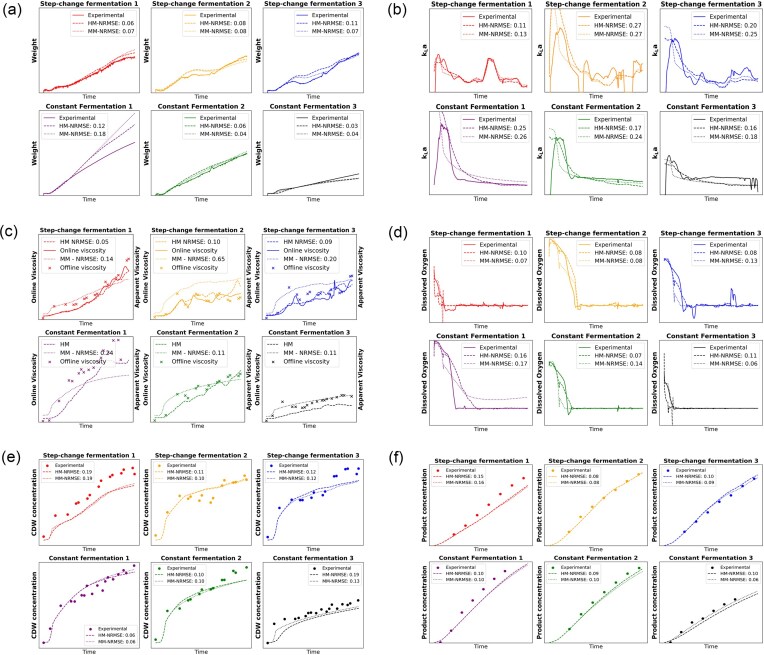
Comparison between the realized fermentation data (continuous lines and dots) and the modeled values (hybrid model [HM]: dashed lines and mechanistic model [MM]: dotted lines) for the six pilot fermentations (550 L).

Therefore, one can combine the mechanistic empirical online *k*_L_*a* correlation and the data-driven online viscosity prediction to create a sequential hybrid model, which can be seen in Figure 8. The model requires a defined user input, which, among others, must include the intended fermentation time, airflow, pressure, agitator speed, and the starting conditions of the model output states as well as the initial online viscosity. The mechanistic model makes use of these inputs and calculates, among others, the nine ML input features (*Y*_1_ to *Y*_9_) that are needed for the LGBM to estimate the online viscosity. Online viscosity is inserted back to the empirical *k*_L_*a* correlation and therefore the mechanistic model. This loop continues until the end of the fermentation is reached. The simulation is executed with an established PID controller, which ensures that a predefined DO profile is maintained by adjusting the feed rate. Figure 9 displays the simulation output for the operation of six different fermentation processes, whose inputs, e.g., agitator speed, pressure, and airflow, are set up as for the actual planned experiments. The top rows are allocated to the three step-change fermentations and the bottom three are the fermentations with three different but constant setpoints for aeration, agitation, and pressure. Additionally, a purely mechanistic model, including a mechanistic prediction of the apparent viscosity, as presented in Albaek et al. (2011), which is fitted to the experimental data in this study, is presented as a comparison and labeled “mechanistic”.

Figure 9 presents predictions of the main fermentation trends from the closed-loop simulation for all six pilot-scale batches and confirms that the prediction accuracy of the developed hybrid model is satisfying over a wide range of operating conditions. Panel B illustrates that the *k*_L_*a* prediction for all six fermentations is overall aligned with the experimental values with NRMSE values between 0.10 and 0.25 for the hybrid and 0.13 and 0.27 for the mechanistic model, respectively. The differences and similarities in the *k*_L_*a* values between the fermentations can be closely linked to the operating conditions that have been applied (Table 1), i.e., the maximum set point for pressure, agitation, and aeration for the first hours for constant fermentation 1 and step change fermentation 2 consequently lead to a similar *k*_L_*a*.

The hybrid online viscosity prediction yields good overall agreement with the batches for which online viscosity data is available. As summarized in Table 1, no online viscosity data is available for the constantly operated fermentation processes. Although the model captures the general trends well, some deviations can be observed in the detailed prediction of the values during step fermentations 2 and 3, as outlined in panel C. The DO prediction displays a stable and matching profile with experimental values for the hybrid model and mechanistic model, except for constant fermentation 1, as seen in panel D. The feed rate of the mechanistic model runs at the maximum rate throughout the entire process and still does not reach the DO target set point due to the high predicted *k*_L_*a* profile. Consequently, the weight mass of this fermentation process is overestimated. The hybrid and mechanistic models demonstrate consistent predictions for CDW and product concentration, as the feed affects the total volume and total amount of CDW or product. Overall, the model captures the experimental trends well; however, toward the end of fermentation, a slight underprediction of CDW values can be observed, as illustrated in panel E. Similarly, the predictions of product concentration largely follow the experimental trends with a slight underprediction, as depicted in panel F.

## Discussion

### Mass transfer correlation

The new proposed “Intra-Batch Experimental Design” to estimate a *k*_L_*a* correlation with reduced experimental effort presents promising results for the online but also offline correlation, based on the *R*^2^ of the correlation fit and NRMSE of the validation with step-change fermentation 3. The comparison between experimentally determined and modeled *k*_L_*a* values demonstrates that it is possible to obtain a reasonable and accurate *k*_L_*a* correlation, with an *R*^2^ of 0.92, with the data points lying within the widely accepted uncertainty range of ±30%, using only two fermentations (Cooke et al., 1988). Step changes in agitation, aeration, and pressure during a fermentation process, as well as the use of online viscosity data, made it possible to examine not only the corner and center points of the design space but also many additional points in between. Therefore, incorporating online viscosity measurements increases the amount of data available for fitting the k_L_*a* correlation by nearly two orders of magnitude. This results not only in significantly lower uncertainty in the fitted parameters (Table 3) but also enhances the intrinsic informativeness and predictive quality of the correlation compared with the offline approach. Moreover, the XL7 probe measures viscosity at a controlled high shear rate (1,200 s⁻¹), which minimizes sensitivity to the heterogeneous shear environment typically found in stirred tank bioreactors and makes the measurement essentially independent of the probe position. Consequently, this high-shear, position-independent online viscosity provides a robust measurement that can be used as input for the *k*_L_*a* correlation.

Validation of the *k*_L_*a* correlation on new data (product A—fermentation 3 in Table 1) indicates good agreement with an NRMSE of 0.12 and 0.07 for the online and offline correlation, respectively. The lower NRMSE observed for the offline correlation, which utilizes offline sample points and apparent viscosity, is therefore unexpected, as this correlation also exhibits a relatively poor fit with an *R*² of 0.67 and larger parameter standard deviations. Nevertheless, since the error remains acceptable for both correlations, it can be concluded that neither model is overfitted with the data from step-change fermentations 1 and 2 (Van’t Riet, 1979). Additionally, the bioreactor system was able to cope with the rather radical step changes. This can be seen by the ability of the controller to keep the DO at the defined DO set point throughout the process, which supports the validity of this new approach. The estimated online exponents (*a* = 0.49, *b* = 0.48, *c* = −0.69) and offline exponents (*a* = 0.52, *b* = 0.35, *c* = −0.81) indicate a slightly different impact of each of the factors toward the OTR coefficient *k*_L_*a*. The newly found values are significantly different from the ones found by Albaek et al. (2011) on a similar process and the conventional approach (*a* = 0.41, *b* = 0.16, *c* = −0.39). Given that the work was carried out in 2011, this comparison does not account for process developments that occurred afterward, such as modifications to media composition. The exponents *a* and *b* from both correlations match the overall ranges that can be found in the literature (0.4 ≤ *a* ≤ 1; 0.3 ≤ *b* ≤ 0.7) (Albaek et al., 2011). The negative influence of the apparent viscosity on *k*_L_*a* is estimated to be −0.81 for the offline correlation and lies outside the range that has been reported so far (−0.4 ≤ *c* ≤ −0.7) (Albaek et al., 2011). The bioprocess model used in this study integrates a *k*_L_*a* correlation, which was originally proposed by Van ’t Riet (1983) and later extended by Cooke et al. (1988) and has since been widely used in bioprocess modeling (Garcia-Ochoa & Gomez, 2009). Despite its broad applicability, this correlation is known to have limitations in terms of parameter identifiability. These identifiability issues affect the interpretability of the estimated parameters and should be considered. The results underscore once again that despite the broad applicability of the general *k*_L_*a* model structure, the specific empirical *k*_L_*a* models themselves might not inherently be suitable for general application (Garcia-Ochoa & Gomez, 2004). Consequently, this highlights the urgent need to minimize the amount of work required to determine these correlations for a specific bioreactor application, as they are inevitable for such a bioprocess model presented in this work.

### Online viscosity model

The validation of the online viscosity models highlights that the LGBM model predicts the online viscosity measurements over all batches most accurately. ML models and especially gradient boost algorithms, where the LBGM belongs to, are known to overfit the data (Ke et al., 2017; Nielsen, 2015). It is therefore crucial to investigate the limits of the model in respect to its predictions, which are addressed in this work by the validation, seen in Figure 7. In more detail, the validation reveals that the model can predict the online viscosity of familiar processes in the pilot reactors to a satisfactory extent. Familiar fermentations are defined as those associated with the product that exists most frequently in the data set (e.g., product A). A decline in prediction accuracy is observed across all online viscosity models for fermentations associated with products represented by only one single fermentation in the training dataset (e.g., product B), as reflected by an increased relative error.

According to the Shapley values (Supplementary Figure S2) (Hammer & Holzman, 1992), the most influential factors impacting the online viscosity prediction are the features: (1) accumulated ammonia flow, (2) accumulated CER, and (3) airflow rate. The first two features provide information about the state, such as cell growth and protein production, and align with the features importance of previous LGBM models on a similar subject (Rydal et al., 2024, Stanbury et al., 2013). The airflow rate, along with the other features, contributes less impactful information to the prediction, which matches the statement from the online viscosity probe manufacturer, Hydramotion Ltd., that operational conditions do not influence the online viscosity probe reading (Hydramotion Ltd., n.d.). Future work could explore whether time-dependent ML models, such as Long Short-Term Memory (LSTM) networks, can improve the performance of this model (Nadda et al., 2024).

### Process simulation

Figure 9 indicates that both the hybrid and fully mechanistic model can predict the course of fermentation that includes a wide range of operational conditions by accurately predicting *k*_L_*a*, with an overall lower NRMSE for the hybrid approach. Furthermore, both models can predict the main fermentation states, such as CDW and product concentration with reasonable accuracy, demonstrating that there is no need to include DO dependency for the growth rate. However, minor deviations occur, which can be attributed to, e.g., the fully experimentally determined yields, which are most likely associated with uncertainties such as measurement errors toward CDW.

It could be argued that the underestimation of *k*_L_*a* for the constant fermentation 3 from both models is caused by a lower viscosity prediction than one would have observed experimentally. The deviation observed in the online viscosity prediction for the three step-change fermentations is most likely due to errors in the predicted input features, which propagate and result in deviations in the online viscosity estimates. Overall, the hybrid model is highly dependent on the accuracy of both its biological and physical components. The accumulation of ammonia or CER, for example, directly affects viscosity estimation, which is linked to the OTR and ultimately the substrate feeding and thus the bioreactor mass that is connected, among others, to all predicted concentrations. Unfortunately, a comparison of the online viscosity prediction for the constant fermentation 1–3 is not possible, as no online viscosity probes were present in these reactors, which makes further statements on such a comparison superfluous. Due to the linear correlation between the growth rate and yields, where the offset of the linear model can be understood as a maintenance term, the bioprocess model takes advantage of varying yields over time. This behavior is observed in experimental data and further supported by the literature, which indicates that cells undergo metabolic changes during fermentation processes, resulting in dynamic biological yields over the course of the fermentation (Van Bodegom, 2007).

The conventional strategy of linking product formation to growth rate in the model, as proposed in the literature due to its association with constitutive promoter expression, cannot capture the linear dynamics of product formation observed in the experimental data (Bandbe et al., 2024; Fitz et al., 2018). Instead, the combination of the production model with a constant rate and the biomass concentration matches the experimental data satisfactorily. A deeper understanding of this could be gained through additional research on the analysis of living cells. Furthermore, the mechanistic component of the model does not include a term for biomass death. This exclusion was based on the lack of reliable and representative measurements about living cells throughout the process, as CDW is not considered as a valid measurement to develop a realistic death term for this fungal industrial process.

Although the hybrid model offers only a modest improvement in prediction accuracy, its primary advantage lies in eliminating the need for offline rheology sampling and the associated challenges in approximating the apparent viscosity. Additionally, incorporating categorical ML input features, such as organism and product type, enables the potential for online viscosity prediction across diverse production strains. In contrast, an apparent viscosity model would require new parameter fitting for each organism and product type. Future work may involve evaluating the model’s performance and the transferability of its development methods to large-scale production. The biological and ML-based viscosity models appear to be well-suited for direct application on a larger scale, while the *k*_L_*a* correlation may need to be examined in more detail.

## Conclusion and outlook

The hybrid model combines a mechanistic approach, stoichiometric details, mass transfer correlations, and ML-based online viscosity predictions to accurately simulate *A. oryzae* pilot scale fermentations over a wide range of operating conditions, achieving a lower NRMSE compared to previous state-of-the-art mechanistic models. Its main advantage, however, is eliminating the need for offline rheology sampling by leveraging online viscosity and categorical inputs such as organism and product type, enabling broader applicability without repeated parameter fitting. In addition, the intra-batch experimental design methodology significantly reduces the number of fermentation processes required while ensuring comprehensive coverage of the design space by utilizing online viscosity data. Alongside the presentation of a hybrid model, this article presents a framework for model development that significantly minimizes both the manual effort required for model construction and the number of fermentations needed. This is an important aspect given the often-limited capacities available at pilot scale. This approach, thus, lowers the costs associated with model development and mitigates potential capacity bottlenecks at pilot scale.

An LGBM model could be utilized and trained to predict online viscosity based on ML inputs calculated from the mechanistic model. This aligns with the performance expectations reported in the literature while also eliminating the need for and the challenges associated with offline viscosity measurements (Grinsztajn et al., 2022; Rydal et al., 2024). However, the model also carries typical limitations due to the use of ML, especially overfitting, which is addressed by using a diverse dataset and by investigation of the model’s performance toward high and low representative fermentations in the training dataset. An even bigger dataset is needed to further explore this aspect. Another promising approach may involve integrating further online data, such as biomass probes for online cell viability measurements during the entire fermentation process (Kiviharju et al., 2008). Adapting both the modeling approach and the model itself to large scales appears promising and should be explored in the future. Altogether, these advances represent a significant step toward transforming model development from a traditionally rather academic and resource-intensive activity into an optimized and more efficient methodology that meets the requirements for industrial standards.

## Supplementary Material

kuag014_Supplemental_Files

## Data Availability

The data underlying this article cannot be shared due to confidentiality obligations arising from industrial collaboration.
