# Supplementary material for: Hybrid modeling for industrial fermentation processes with an “Intra-Batch Experimental Design”
Source: J Ind Microbiol Biotechnol. 2026 Jun 2;53:kuag014. doi: 10.1093/jimb/kuag014 (PMC13278493; doi:10.1093/jimb/kuag014)
Supplement: kuag014_Supplemental_Files [file kuag014_supplemental_files.zip › Sup_Infos_Hybrid Modeling_revised.docx]

Supporting Information:

# Linear correlation between growth rate and each respective yield.

| 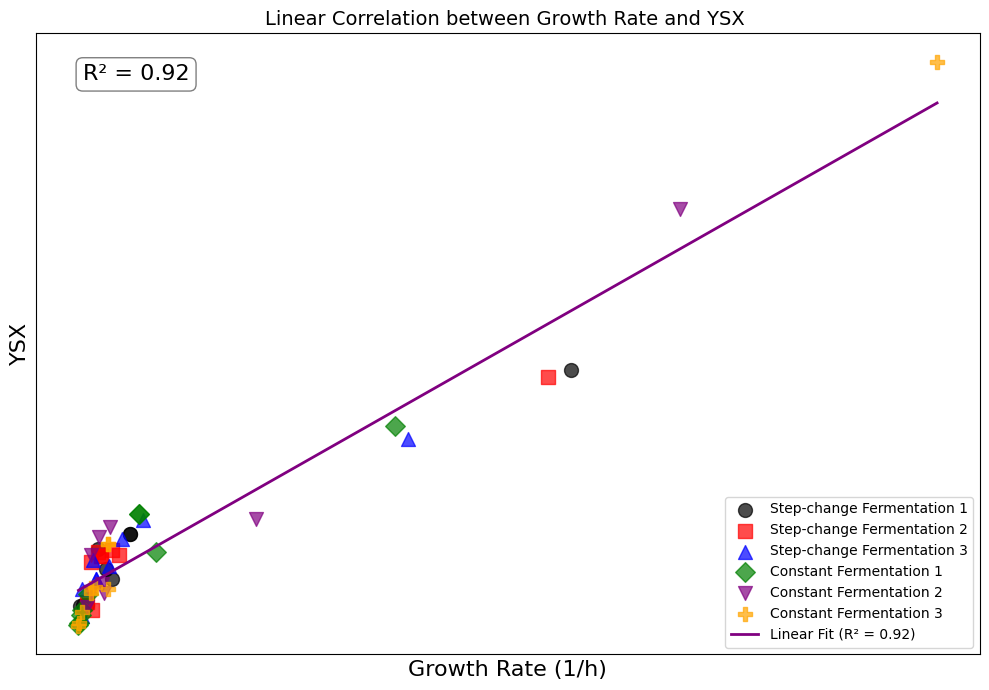 | 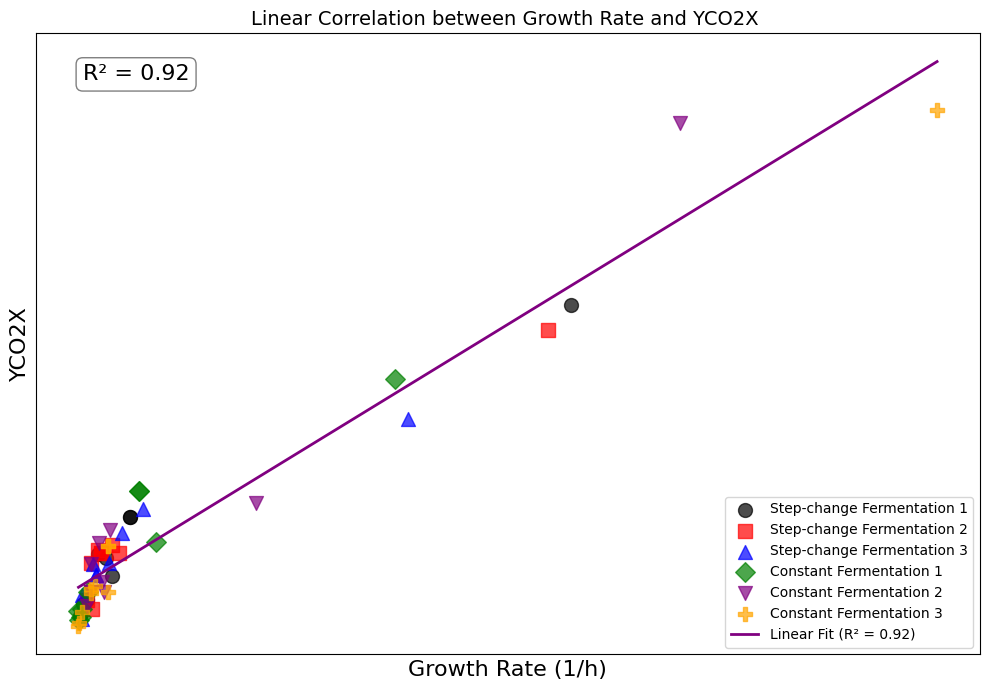 |
| --- | --- |
| 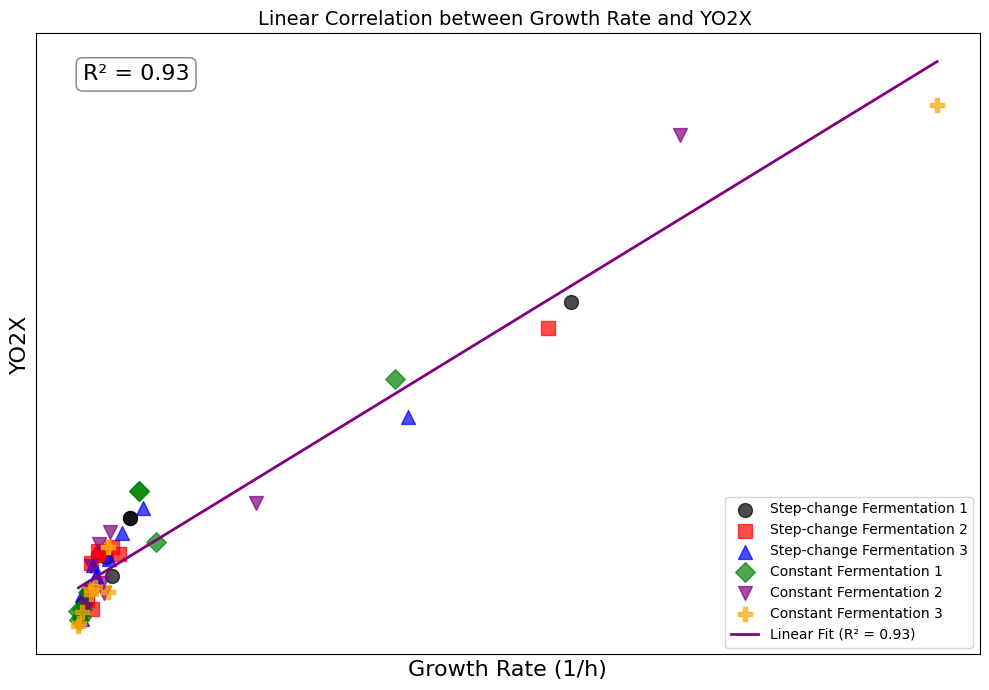 | 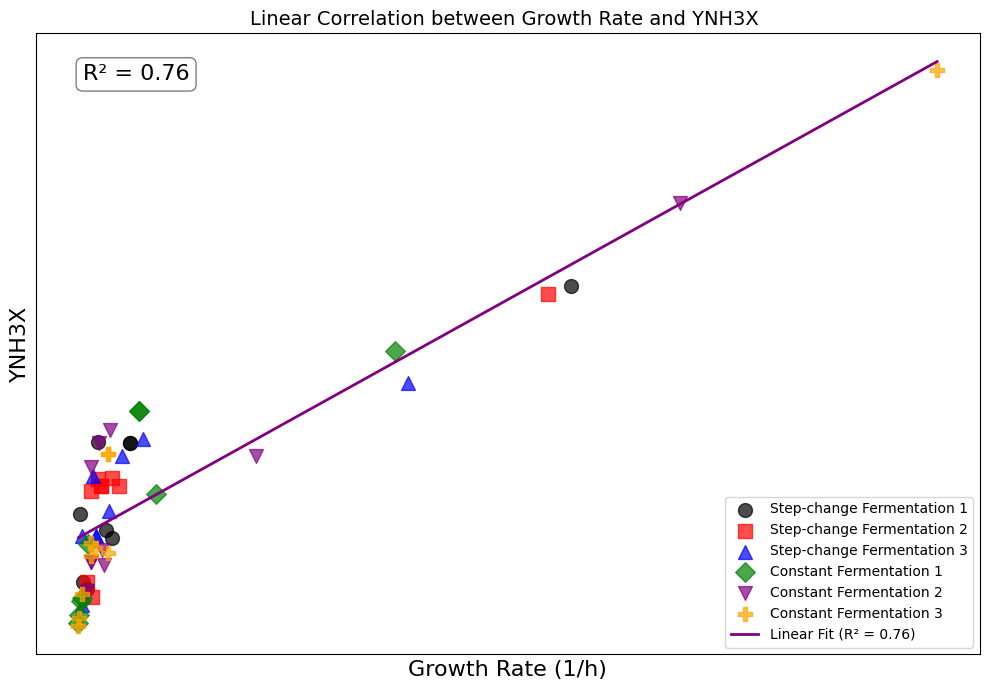 |

Figure S1: Linear regression model between the transient yield and the growth rate in respect to Step-change fermentation 1 and 2. The actual linear regression model cannot be shown for confidentiality reasons.

# SHAP values, Residual analysis and cross validation score of the LGBM:


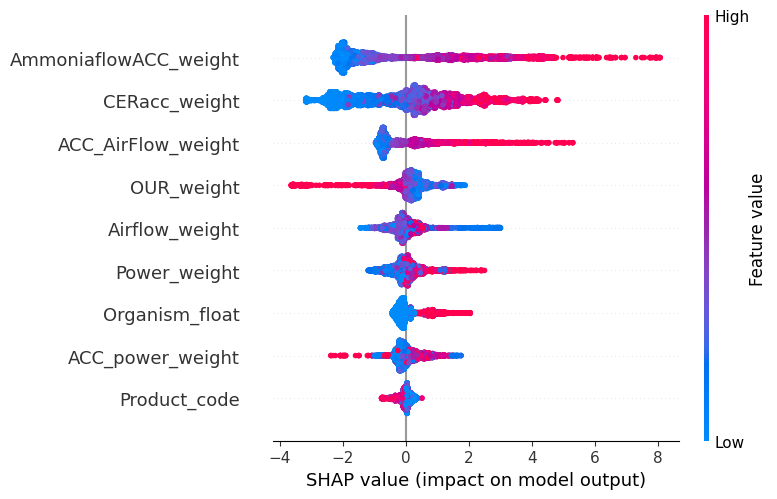


| Figure S2: SHAPly values of the LGBM model |  |
| --- | --- |

Cross validation score between all fermentation batches: R²: 0.834


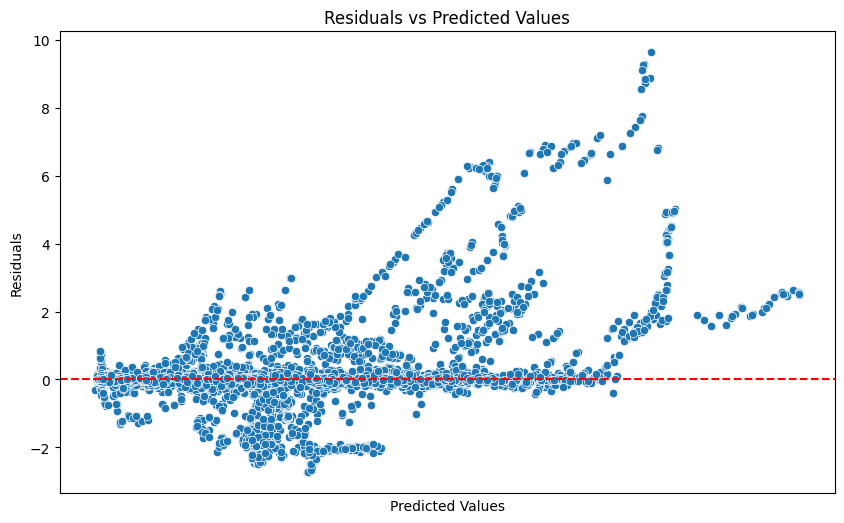


Figure S3: Residual analysis for the training dataset

Table S1: Overview: Hyperparameter and Grid search, optimization ranges for the LGBM, ANN and PLS

| **Parameter** | **LGBM - value** | **LGBM - range** | **ANN - value** | **ANN - grid** | **PLS - value** |
| --- | --- | --- | --- | --- | --- |
| boosting_type | gbdt |  |  |  |  |
| class_weight | null |  |  |  |  |
| colsample_bytree | 0.932 | 0.7-1.0 |  |  |  |
| importance_type | split |  |  |  |  |
| learning_rate | 0.090 | 0.01-0.4 | "constant" |  |  |
| logging_level | error |  |  |  |  |
| max_depth | 5 | 5.0-12 |  |  |  |
| min_child_samples | 15 | 0-20 |  |  |  |
| min_child_weight | 0.001 |  |  |  |  |
| min_split_gain | 0.0 |  |  |  |  |
| n_estimators | 1352 | 50-2000 |  |  |  |
| n_jobs | -1 |  |  |  |  |
| num_leaves | 31 |  |  |  |  |
| objective | null |  |  |  |  |
| random_state | 42 |  | 42 |  |  |
| reg_alpha | 0.0685 | 0.0-2 |  |  |  |
| reg_lambda | 0.2445 |  |  |  |  |
| subsample | 0.6501 | 0.6-1.0 |  |  |  |
| subsample_for_bin | 200000 |  |  |  |  |
| subsample_freq | 0 |  |  |  |  |
| verbose | -1 |  | false |  |  |
| activation |  |  | tanh | [relu, tanh] |  |
| alpha |  |  | 0.0001 | [0.0001, 0.001] |  |
| batch_size |  |  | auto |  |  |
| beta_1 |  |  | 0.9 |  |  |
| beta_2 |  |  | 0.999 |  |  |
| early_stopping |  |  | true | [true] |  |
| epsilon |  |  | 1e-08 |  |  |
| hidden_layer_sizes |  |  | [64, 32] | [[64], [64, 32], [100, 50]] |  |
| learning_rate_init |  |  | 0.01 | [0.001, 0.01] |  |
| max_fun |  |  | 15000 |  |  |
| max_iter |  |  | 500 | [500] | 500 |
| momentum |  |  | 0.9 |  |  |
| n_iter_no_change |  |  | 20 | [20] |  |
| nesterovs_momentum |  |  | true |  |  |
| power_t |  |  | 0.5 |  |  |
| shuffle |  |  | true |  |  |
| solver |  |  | adam | [adam] |  |
| tol |  |  | 0.0001 |  | 1e-06 |
| validation_fraction |  |  | 0.1 |  |  |
| warm_start |  |  | false |  |  |
| copy |  |  |  |  | true |
| n_components |  |  |  |  | 7 |
| scale |  |  |  |  | true |

1. **Overview of fermentations from the machine learning dataset**

Table S2: Overview of the fermentation batches, whose data was used to develop the machine learning model.

| Name | Product | Fermentation | Production scale or Pilot scale | Training or ***Validation*** of the mode |
| --- | --- | --- | --- | --- |
| Product A - Step-change fermentation 3 | A | 1 | Pilot scale | ***Validation*** |
| Product A - Step-change fermentation 1 | A | 2 | Pilot scale | Training |
| Product A - Step-change fermentation 2 | A | 3 | Pilot scale | Training |
| Product A - Fermentation 4 | A | 4 | Pilot scale | Training |
| Product A - Fermentation 5 | A | 5 | Pilot scale | Training |
| Product A - Fermentation 6 | A | 6 | Pilot scale | Training |
| Product A - Fermentation 7 | A | 7 | Pilot scale | Training |
| Product A - Fermentation 8 | A | 8 | Pilot scale | ***Validation*** |
| Product A - Fermentation 9 | A | 9 | Pilot scale | Training |
| Product A - Fermentation 10 | A | 10 | Pilot scale | Training |
| Product A - Fermentation 11 | A | 11 | Pilot scale | Training |
| Product B - Fermentation 1 | B | 1 | Production scale | ***Validation*** |
| Product B - Fermentation 2 | B | 2 | Production scale | Training |
| Product C - Fermentation 1 | C | 1 | Production scale | Training |
| Product D - Fermentation 1 | D | 1 | Production scale | Training |
| Product D - Fermentation 2 | D | 2 | Production scale | Training |
| Product E - Fermentation 1 | E | 1 | Production scale | Training |
| Product E - Fermentation 2 | E | 2 | Production scale | Training |
| Product E - Fermentation 3 | E | 3 | Production scale | Training |
| Product E - Fermentation 4 | E | 4 | Production scale | Training |
| Product E - Fermentation 5 | E | 5 | Production scale | Training |
| Product F - Fermentation 1 | F | 1 | Pilot scale | Training |
| Product F - Fermentation 2 | F | 2 | Pilot scale | Training |
| Product G - Fermentation 1 | G | 1 | Pilot scale | Training |
| Product H - Fermentation 1 | H | 1 | Pilot scale | Training |
| Product I - Fermentation 1 | I | 1 | Pilot scale | Training |
| Product J - Fermentation 1 | J | 1 | Pilot scale | Training |
| Product K - Fermentation 1 | K | 1 | Pilot scale | Training |
| Product L - Fermentation 1 | L | 1 | Pilot scale | Training |
| Product M - Fermentation 1 | M | 1 | Pilot scale | Training |


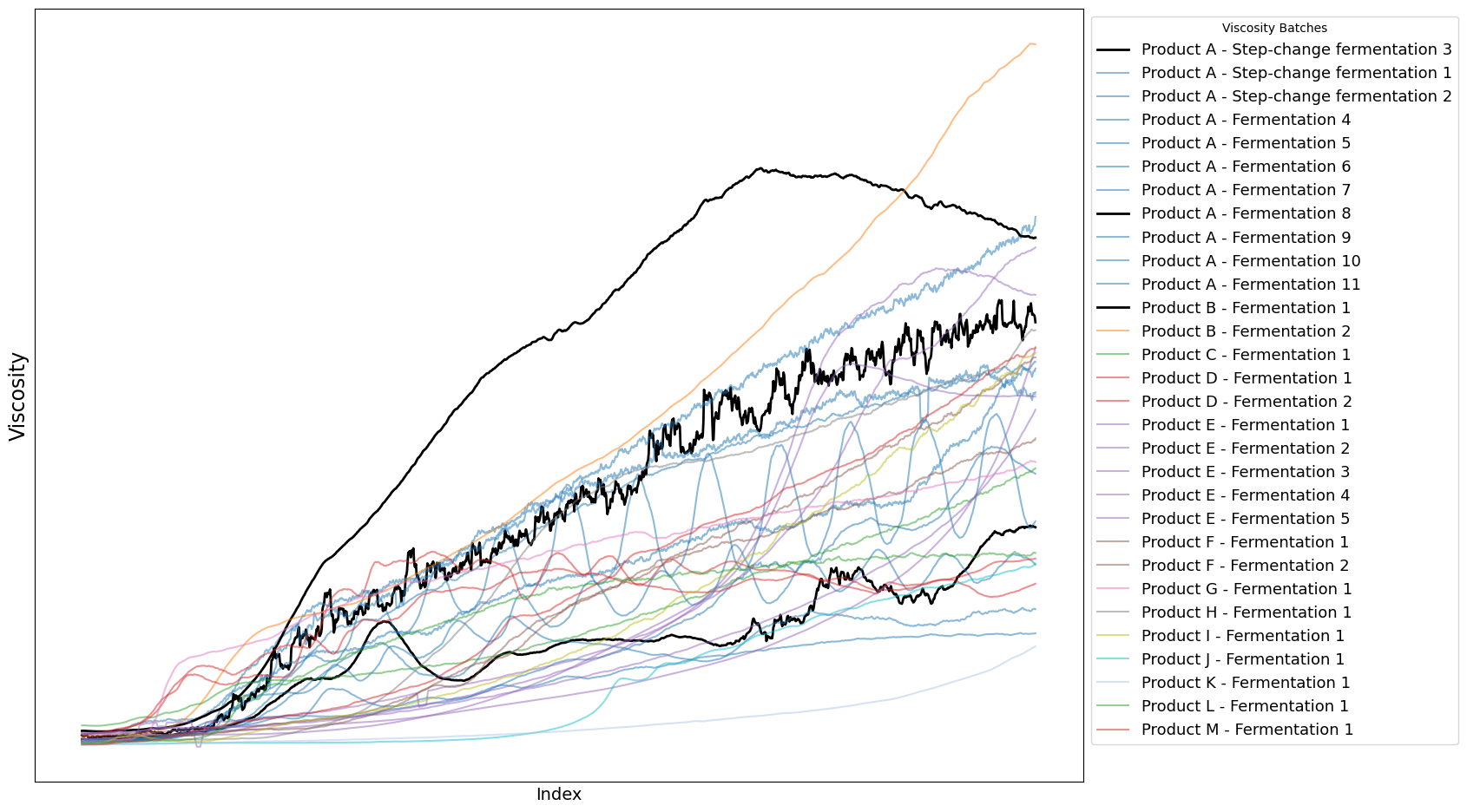


Figure S4: All colorful batches have been used for the model training. The black viscosity profiles denote those batches, on which the viscosity model was validated and tested after the training phase.
