# Supplementary figures and images for: Hybrid modeling for industrial fermentation processes with an “Intra-Batch Experimental Design”
Source: J Ind Microbiol Biotechnol. 2026 Jun 2;53:kuag014. doi: 10.1093/jimb/kuag014 (PMC13278493; doi:10.1093/jimb/kuag014)

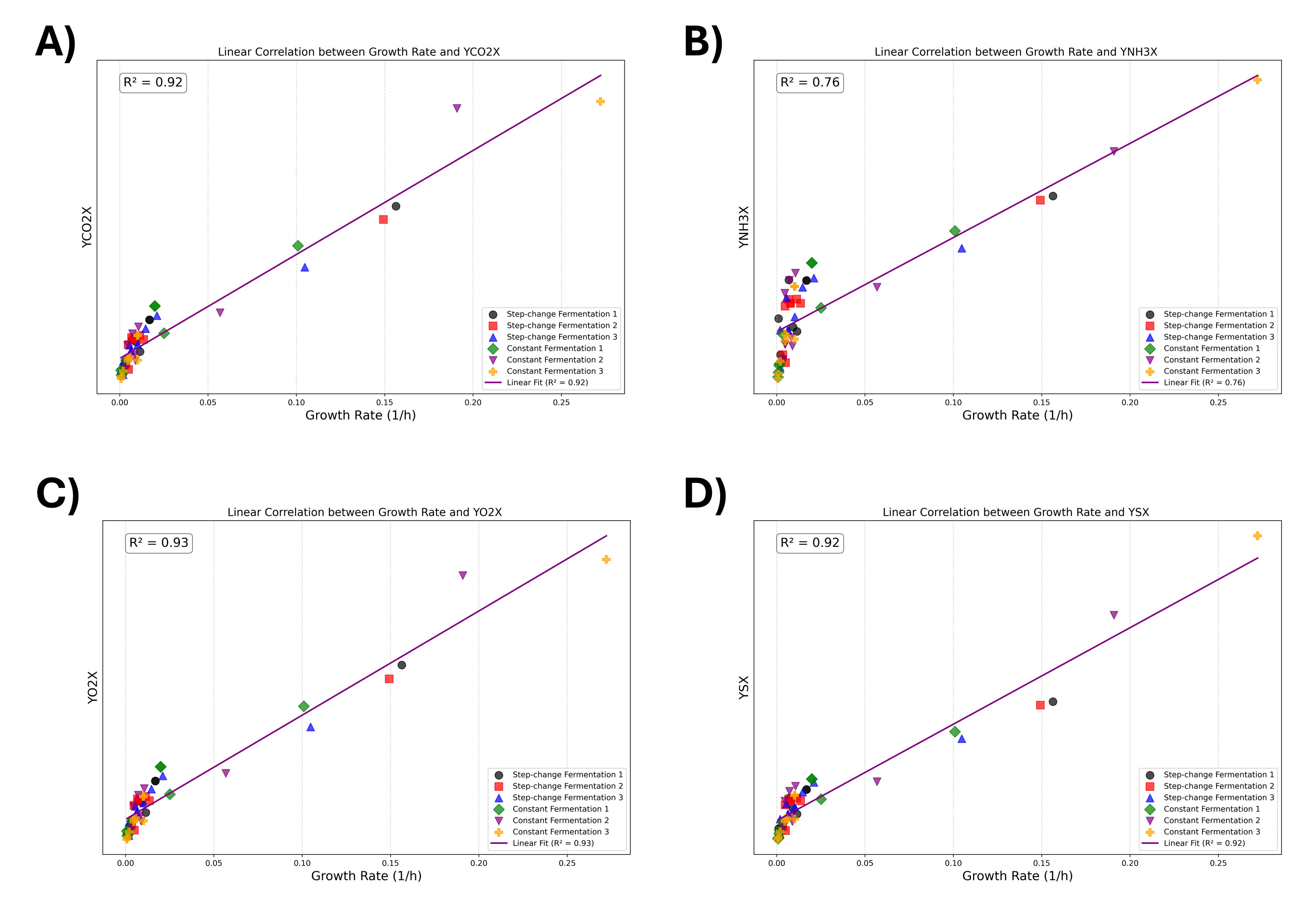

Supplement: kuag014_Supplemental_Files [file kuag014_supplemental_files.zip › figureS1_grouped.tif]

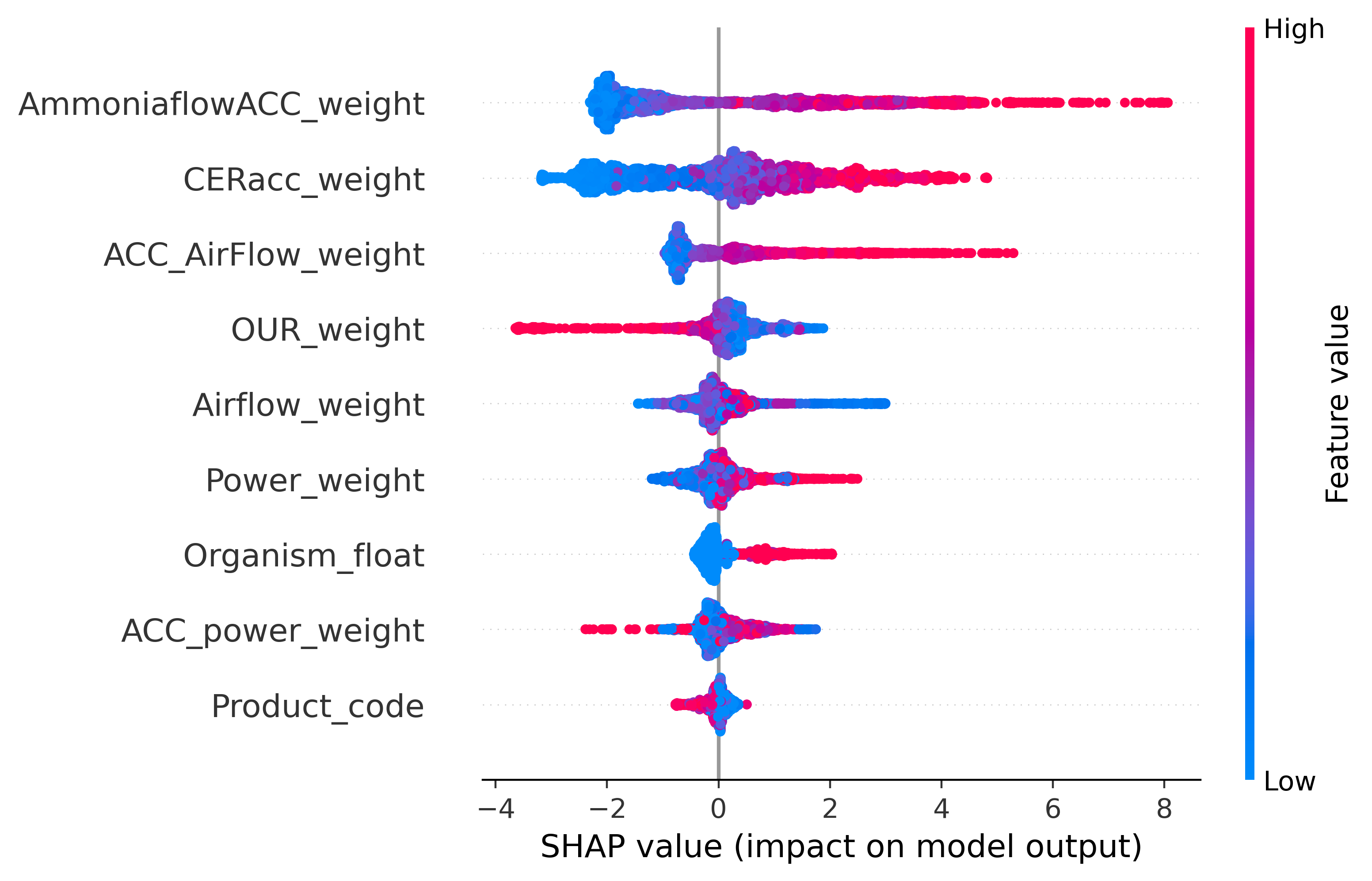

Supplement: kuag014_Supplemental_Files [file kuag014_supplemental_files.zip › FigureS2.png]

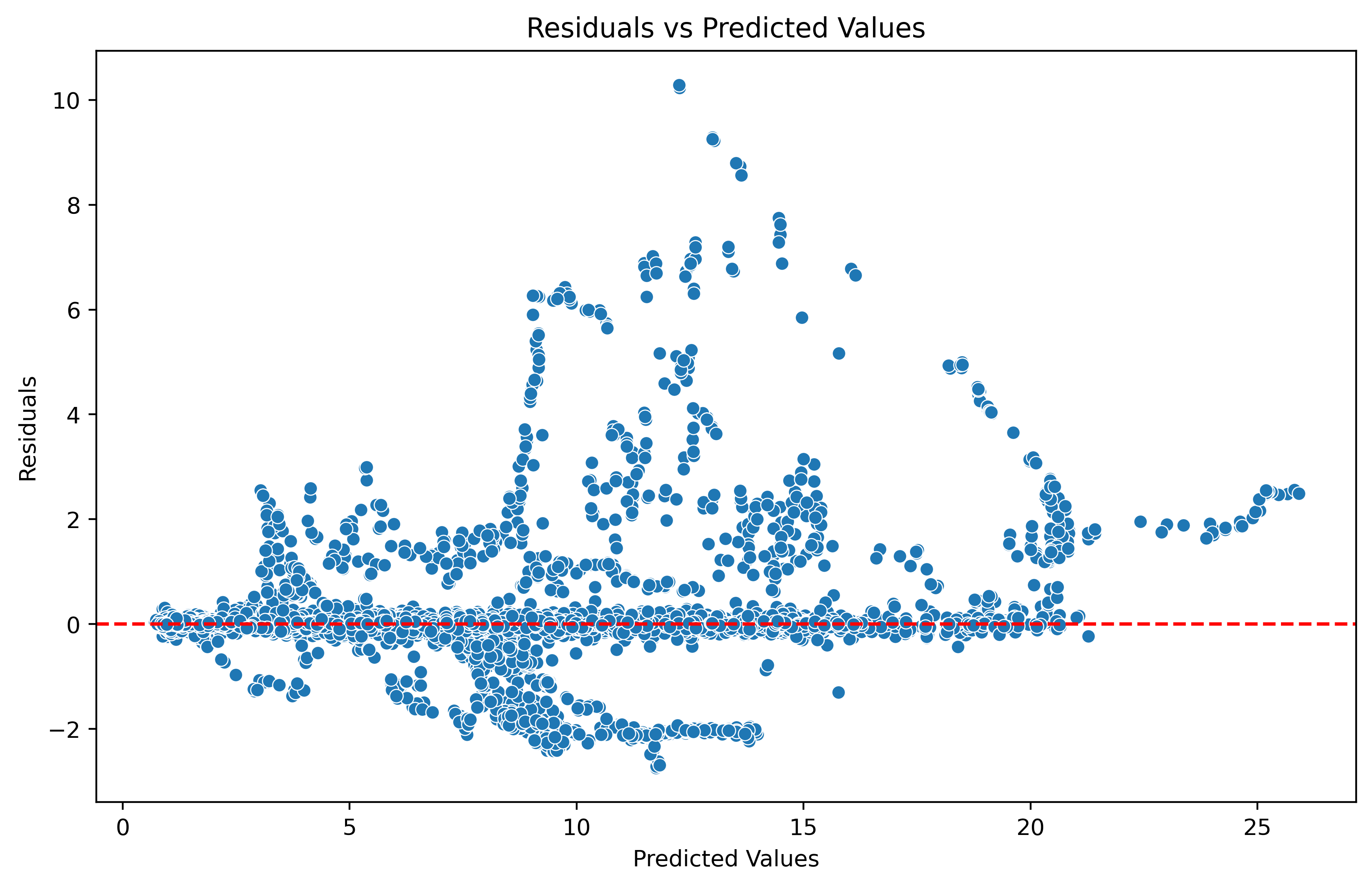

Supplement: kuag014_Supplemental_Files [file kuag014_supplemental_files.zip › FigureS3.png]

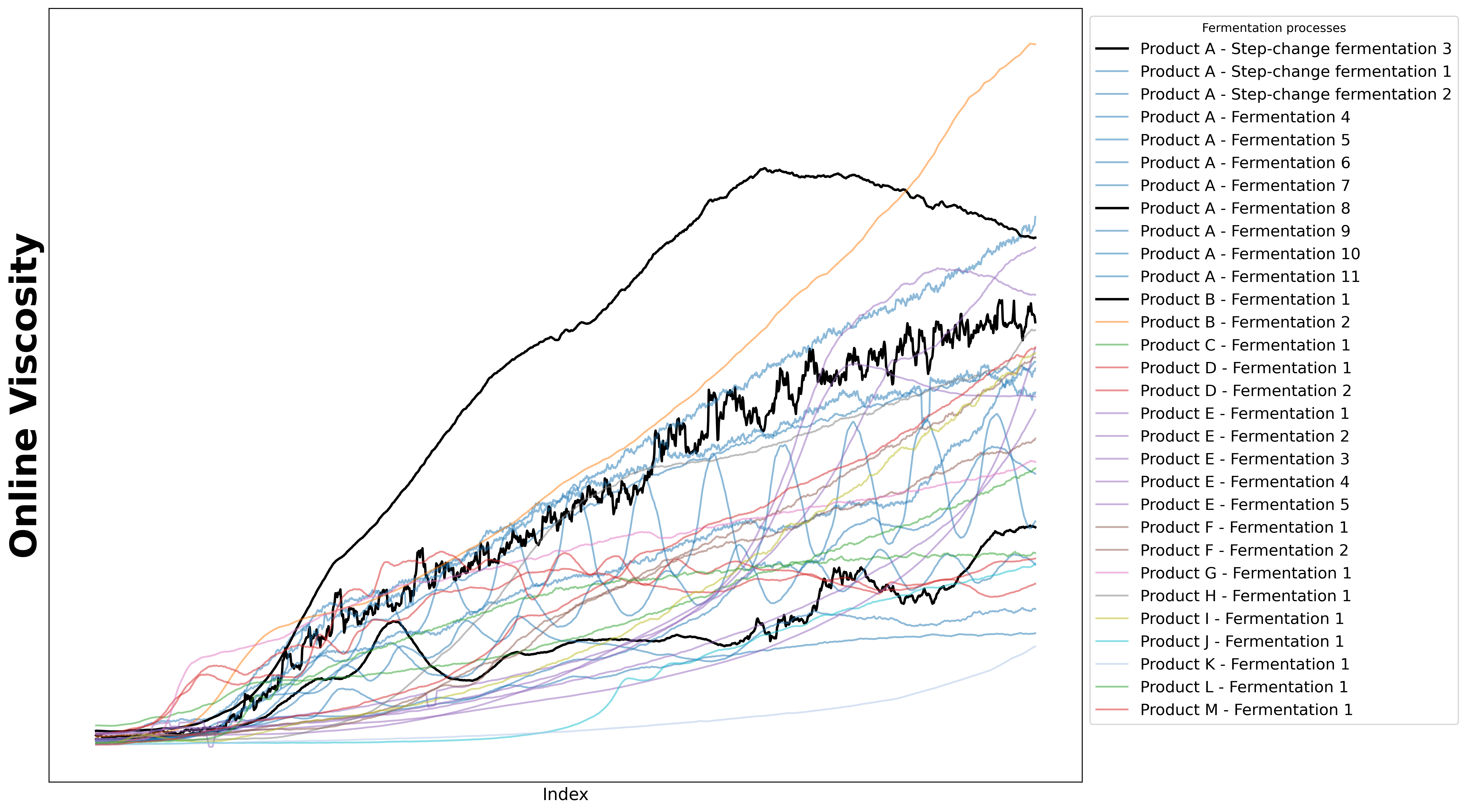

Supplement: kuag014_Supplemental_Files [file kuag014_supplemental_files.zip › FigureS4.png]
